# The multifaceted consequences and economic costs of child anxiety problems: A systematic review and meta‐analysis

**DOI:** 10.1002/jcv2.12149

**Published:** 2023-04-21

**Authors:** Jack Pollard, Tessa Reardon, Chloe Williams, Cathy Creswell, Tamsin Ford, Alastair Gray, Nia Roberts, Paul Stallard, Obioha C. Ukoumunne, Mara Violato

**Affiliations:** ^1^ Health Economics Research Centre Nuffield Department of Population Health University of Oxford Oxford UK; ^2^ Departments of Experimental Psychology and Psychiatry University of Oxford Oxford UK; ^3^ University of Cambridge and Cambridge and Peterborough Foundation Trust Cambridge UK; ^4^ Bodleian Health Care Libraries University of Oxford Oxford UK; ^5^ Department of Health University of Bath Bath UK; ^6^ NIHR Applied Research Collaboration South West Peninsula (PenARC) University of Exeter Exeter UK

**Keywords:** anxiety, children, economic cost, meta‐analysis, outcomes, systematic review

## Abstract

**Background:**

Over a quarter of people have an anxiety disorder at some point in their life, with many first experiencing difficulties during childhood or adolescence. Despite this, gaps still exist in the current evidence base of the multiple consequences of childhood anxiety problems and their costs.

**Methods:**

A systematic review of Medline, PsycINFO, EconLit and the National Health Service Economic Evaluation Database was conducted for longitudinal and economic studies reporting on the association between childhood anxiety problems and at least one individual‐, family‐ or societal‐level outcome or cost. All studies were synthesised narratively. For longitudinal studies, ‘effect direction’ was used as a common metric, with random effects meta‐analysis undertaken where possible.

**Results:**

Eighty‐three studies met inclusion criteria and were synthesised narratively. We identified 788 separate analyses from the longitudinal studies, which we grouped into 15 overarching outcome domains. Thirteen of the studies were incorporated into 13 meta‐analyses, which indicated that childhood anxiety disorders were associated with future anxiety, mood, behaviour and substance disorders. Narrative synthesis also suggested associations between anxiety problems and worse physical health, behaviour, self‐harm, eating, relationship, educational, health care, employment, and financial outcomes. ‘Effect direction’ was conflicting in some domains due to a sparse evidence base. Higher economic costs were identified for the child, their families, healthcare providers and wider society, although evidence was limited and only covered short follow‐up periods, up to a maximum of 2 years. Total annual societal costs per anxious child were up to £4040 (2021 GBP).

**Conclusions:**

Childhood anxiety problems are associated with impaired outcomes in numerous domains, and considerable economic costs, which highlight the need for cost‐effective interventions and policies to tackle them. More economic evidence is needed to inform models of the long‐term, economic‐related, consequences of childhood anxiety problems.

## Graphical abstract

In this systematic review, we provide a holistic overview of the consequences of anxiety problems experienced in childhood. We found that most of the available evidence primarily investigated health‐related consequences, mainly at the individual child‐level, and overlooked the wider impacts on families and society at large. Measuring these wider impacts is vital forinforming multidisciplinary policies and interventions aimed at tackling childhood problems and their consequences, and more research in these areas is therefore warranted.

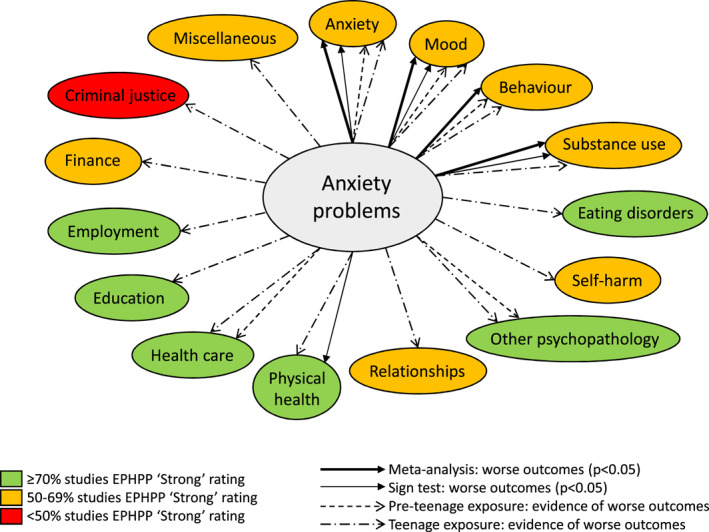

**Key points**

- Despite high prevalence, gaps still exist in the current evidence base of the multiple consequences of childhood anxiety problems and their costs.
- This systematic review is the first to synthesise the evidence across the multiple consequences of childhood anxiety problems, including the associated economic costs.
- Childhood anxiety problems often persist into adulthood and are associated with worse outcomes in numerous areas of life, from mental health to physical health and educational and employment outcomes, as well as higher societal economic costs.
- The holistic overview of the impact of childhood anxiety at the child, family and societal level provides vital information to clinicians and other stakeholders for informing decisions on approaches to prevent and treat childhood anxiety problems.

## INTRODUCTION

Anxiety disorders are common; 29% of people meet diagnostic criteria at some point in their life (Kessler et al., 2005). Many common anxiety disorders are first experienced during childhood or adolescence (Solmi et al., 2021), with at least one in every 15 children affected at any one time (Polanczyk et al., 2015). Anxiety disorders were reported to be the ninth leading cause of years lived with disability (YLD) in 2016 (Vos et al., 2017). Annually, anxiety disorders account for 5.7 million YLD in young people under 20 years worldwide, making up 4.3% of total YLD in this age group (Vos et al., 2020). The high prevalence and impairment associated with anxiety disorders result in the fifth highest economic burden of all brain disorders, almost half of which are indirect costs (e.g. lost productivity due to missed work) (Fineberg et al., 2013). When focussing on children specifically, the societal cost of anxiety disorders is estimated to be 21 times greater than children without the disorder (Bodden, Dirksen, & Bogels, 2008).

Empirical evidence exists on the association between childhood anxiety disorders and numerous consequences. Firstly, childhood anxiety disorders are associated with various individual‐level outcomes, including a greater risk of developing other mental health, substance use and eating disorders (Essau et al., 2014; Shevlin et al., 2017; Sihvola et al., 2009), and self‐harm (Mars et al., 2019). Moreover, they are also associated with poorer physical health (Chen et al., 2009), education attainment (Dalsgaard et al., 2020), and employment outcomes in later life (Knapp et al., 2011). Secondly, anxiety disorders in childhood are related to various family‐level outcomes, such as poorer parent‐reported family functioning (Towe‐Goodman et al., 2014), caregivers missing work (Pella et al., 2020; van Steensel, Dirksen, & Bogels, 2013) and providing informal care (Bodden, Dirksen, & Bogels, 2008). Finally, childhood anxiety disorders also affect societal‐level outcomes. For example, childhood anxiety disorders were linked to higher levels of health care use (Ali et al., 2018), and wider consequences, such as additional educational support during school age, and lost productivity among parents missing work (Pella et al., 2020, van Steensel et al., 2013).

Multiple literature reviews have explored the association between childhood anxiety problems and specific outcomes, such as depression (Schleider et al., 2014), attention‐deficit/hyperactivity disorder (ADHD) (D'Agati et al., 2019), substance use (Groenman et al., 2017), anorexia nervosa (Lloyd et al., 2019) and school attendance (Finning et al., 2019). These individual studies are valuable when addressing specific research and clinical questions, but less useful in identifying and quantifying the multidimensional consequences of childhood anxiety problems across the life course. A holistic overview of the impact of child anxiety problems at the child, family and societal level is vital for informing multidisciplinary policies and interventions aimed at tackling these problems and their consequences. This approach also provides a comprehensive overview for those without specialist knowledge of the topic (for example, health economists/modellers, policy‐makers), as well as additional insight, beyond their typical area of expertise, for those with specialist knowledge of specific outcome areas. Furthermore, synthesising this evidence in a single study provides rigorous and readily available data to inform economic models to predict the long‐term outcomes of childhood anxiety problems, an area that is insufficiently researched, supporting in this way the economic case for preventative and early intervention measures to tackle these common childhood problems. Finally, our holistic approach enables conclusions to be drawn about the depth of evidence that exists in different outcome areas, allowing well informed recommendations to be made about where future research would be best focussed.

To our knowledge, no systematic review to date has synthesised the evidence across the multiple consequences of childhood anxiety problems. In this study, we undertook a comprehensive systematic rapid review and meta‐analysis to establish the multifaceted outcomes of child anxiety problems, and the associated economic costs.

## METHODS

The rapid systematic review was registered on PROSPERO (CRD42021202440) (Pollard et al., 2021) and was conducted and reported in line with best practice guidelines (Garritty et al., 2021; Moher et al., 2009).

### Eligibility criteria

Studies were included if they met the following criteria: English language peer‐reviewed publications from 2000 onwards; focussed on high‐income countries; anxiety problem exposure in childhood or adolescence (≤18 years); reported at least one quantifiable individual‐, family‐ or societal‐level outcome associated with the exposure; longitudinal design or economic study (i.e., economic evaluation, costing or burden of disease study). For this review, we define child anxiety problems as the presence of an anxiety disorder or elevated anxiety symptoms under 19 years, on the basis of (a) a Diagnostic and Statistical Manual of Mental Disorders (DSM)‐5 or ICD‐10 code; (b) a diagnosis using a gold standard semi‐structured interview or from a trained professional; or (c) an established cut‐off score on a validated questionnaires. Appendix S1 reports full definitions and detailed inclusion/exclusion criteria.

### Identification of studies

Medline (OvidSP) [1946‐present], PsycINFO (OvidSP) [1806‐present], EconLit (Proquest) and the National Health Service Economic Evaluation Database [https://www.crd.york.ac.uk/CRDWeb/] were searched on 3^rd^ July 2020, and re‐searched on 16^th^ March 2021, for studies published in English from 2000 onwards, using the strategy outlined in Appendix S2. Results were exported to Endnote 20 for deduplication.

A randomly selected 20% sample of studies was double screened on title/abstract and full text by Chloe Williams (CW) and Jack Pollard (JP) to ensure consistency. CW and JP achieved 85% and 87% agreement with the title/abstract and full text double screening respectively, with disagreements resolved by consensus with Mara Violato (MV). The remainder were shared for single screening. Reasons for exclusion were documented at full text review. Reference lists of included studies were screened to identify relevant literature that may have been missed in the search strategy. Corresponding authors were approached for the full text of articles that could not be accessed. Review team members also contributed important papers that were not identified by the search strategy. Advice on specific clinical‐related queries was obtained from clinical team members where necessary.

### Data extraction

Data extraction forms were tailored to study design (longitudinal or economic study), and finalised after piloting. Specific data extraction categories are reported in Appendix S3.

### Quality assessment

Quality was assessed using the Effective Public Health Practice Project (EPHPP) ‘Quality assessment tool for quantitative studies’ (Thomas et al., 2003), which is applicable across study designs and is recommended by the Cochrane Public Health Group (Armijo‐Olivo, Stiles, Hagen, Biondo, & Cummings, 2012; Landeiro et al., 2018). Economic evaluations were also assessed for reporting quality using the Consolidated Health Economic Evaluation Reporting Standards (CHEERS) checklist (Husereau et al., 2013).

Data extraction and quality assessment was completed by CW and JP for a 20% sample of studies, to ensure consistency, with the remainder shared between the two for single extraction/assessment.

### Data synthesis

Methodological heterogeneity between studies prohibited statistical synthesis of findings across all studies together, so we synthesised findings narratively, with meta‐analysis undertaken where possible (see below). Given differences in design and outcomes between the longitudinal and economic studies, they were synthesised separately.

#### Longitudinal studies

##### Narrative synthesis

Firstly, a narrative synthesis was undertaken. We grouped outcomes that were conceptually similar, but measured in different ways, in 15 overarching, mutually exclusive, outcome domains. These were anxiety (e.g. social anxiety disorder), mood (e.g. depressive disorder), behaviour (e.g. disruptive disorder), substance use (e.g. substance use disorder (SUD)), eating (e.g. anorexia nervosa), self‐harm (e.g. suicide attempt), other psychopathology (e.g. psychosis, schizophreniform disorders), relationships (e.g. interpersonal functioning), physical health (e.g. body mass index), health care use (e.g. mental health care visits), education (e.g. final exam grade), employment (e.g. occupation), finance (e.g. financial functioning), criminal justice (e.g. criminal offences), and miscellaneous (e.g. self‐esteem). A full list of individual outcomes included in each domain can be found in Appendix S3.

Synthesis was undertaken through vote counting based on direction of effect, a standardised metric to synthesise diverse outcomes and effect measures across studies, as recommended in the Cochrane Handbook for Systematic Reviews of Interventions (McKenzie & Brennan, 2022). The approach assesses whether the evidence indicates an improvement, worsening or no clear change in each outcome of interest, regardless of the statistical significance of the association. It is appropriate when reviews incorporate non‐randomised studies and there is inconsistency in the effect measures across studies, due to the various study designs and outcomes included (Boon & Thomson, 2021; McKenzie & Brennan, 2022). Firstly, for each outcome domain, we categorised all exposure‐outcome associations, extracted across all studies, into three mutually exclusive categories: (a) outcome worsened; (b) no change in outcome (i.e., OR/incidence rate ratio/hazard ratio = 1); (c) outcome improved. Exposure was classified as ‘pre‐teenage’ (0–12 years) or ‘teenage’ (13–18 years), and outcomes as ‘pre‐teenage’, ‘teenage’, or ‘adult’ (≥19 years), based on sample average age where provided, or the upper limit of the age range otherwise (Table 1 and Figure S1).

**TABLE 1:** Number (%) of reported associations and strength of evidence between anxiety exposure and each outcome domain across studies.

Outcome	Pre‐teenage exposure					Teenage exposure					Overall	
	Studies	Analyses	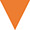	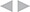	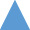	Studies	Analyses	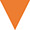	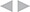	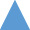	Studies	Analyses
**Anxiety**	**10**	**92**	**78 (85%)**	**3 (3%)**	**11 (12%)**	**18**	**89**	**75 (84%)**	**–**	**14 (16%)**	**25**	**181**
Pre‐teen outcome	3	19	17 (89%)	–	2 (11%)	∼	∼	∼	∼	∼	3	19
Teen outcome	3	14	14 (100%)	–	–	8	38	31 (82%)	–	7 (18%)	11	52
Adult outcome	4	59	47 (80%)	3 (5%)	9 (15%)	11	51	44 (86%)	–	7 (14%)	13	110
**Mood**	**11**	**24**	**20 (83%)**	**1 (4%)**	**3 (13%)**	**20**	**61**	**57 (93%)**	**–**	**4 (7%)**	**28**	**85**
Pre‐teen outcome	5	8	7 (88%)	–	1 (13%)	∼	∼	∼	∼	∼	5	8
Teen outcome	3	7	7 (100%)	–	–	10	23	21 (91%)	–	2 (9%)	13	30
Adult outcome	3	9	6 (67%)	1 (11%)	2 (22%)	12	38	36 (95%)	–	2 (5%)	13	47
**Behaviour**	**4**	**21**	**18 (86%)**	**1 (5%)**	**2 (10%)**	**5**	**9**	**3 (33%)**	**2 (22%)**	**4 (44%)**	**8**	**30**
Pre‐teen outcome	1	3	3 (100%)	–	–	∼	∼	∼	∼	∼	1	3
Teen outcome	1	12	12 (100%)	–	–	2	3	2 (67%)	1 (33%)	–	3	15
Adult outcome	2	6	3 (50%)	1 (17%)	2 (33%)	3	6	1 (17%)	1 (17%)	4 (67%)	4	12
**Substance use**	**7**	**53**	**29 (55%)**	**1 (2%)**	**23 (43%)**	**20**	**178**	**125 (70%)**	**5 (3%)**	**48 (27%)**	**24**	**231**
Pre‐teen outcome	0	0	–	–	–	∼	∼	∼	∼	∼	0	0
Teen outcome	4	43	23 (53%)	1 (2%)	19 (44%)	6	20	12 (60%)	2 (10%)	6 (30%)	10	63
Adult outcome	3	10	6 (60%)	‐	4 (40%)	14	158	113 (72%)	3 (2%)	42 (27%)	15	168
**Eating disorders**	**0**	**0**	**–**	**–**	**–**	**4**	**21**	**16 (76%)**	**–**	**5 (24%)**	**4**	**21**
Pre‐teen outcome	0	0	–	–	–	∼	∼	∼	∼	∼	0	0
Teen outcome	0	0	–	–	–	4	21	16 (76%)	–	5 (24%)	4	21
Adult outcome	0	0	–	–	–	0	0	–	–	–	0	0
**Self‐harm**	**1**	**1**	**–**	**–**	**1 (100%)**	**4**	**17**	**17 (100%)**	**–**	**–**	**4**	**18**
Pre‐teen outcome	0	0	–	–	–	∼	∼	∼	∼	∼	0	0
Teen outcome	0	0	–	–	–	2	9	9 (100%)	–	–	2	9
Adult outcome	1	1	–	–	1 (100%)	2	8	8 (100%)	–	–	2	9
**Other psychology**	**3**	**6**	**4 (67%)**	–	**2 (33%)**	**8**	**16**	**12 (75%)**	–	**4 (25%)**	**9**	**22**
Pre‐teen outcome	1	1	–	–	1 (100%)	∼	∼	∼	∼	∼	1	1
Teen outcome	1	4	4 (100%)	–	–	2	4	2 (50%)	–	2 (50%)	3	8
Adult outcome	1	1	‐	–	1 (100%)	6	12	10 (83%)	–	2 (17%)	6	13
**Relationships**	**2**	**10**	**2 (20%)**	**–**	**8 (80%)**	**5**	**34**	**28 (82%)**	**2 (6%)**	**4 (12%)**	**6**	**44**
Pre‐teen outcome	1	2	2 (100%)	–	–	∼	∼	∼	∼	∼	1	2
Teen outcome	0	0	–	–	–	2	21	17 (81%)	2 (10%)	2 (10%)	2	21
Adult outcome	1	8	–	–	8 (100%)	3	13	11 (85%)	–	2 (15%)	3	21
**Physical health**	**4**	**30**	**17 (57%)**	**–**	**13 (43%)**	**7**	**27**	**23 (85%)**	**1 (4%)**	**3 (11%)**	**10**	**57**
Pre‐teen outcome	0	0	–	–	–	∼	∼	∼	∼	∼	0	0
Teen outcome	3	29	16 (55%)	–	13 (45%)	2	15	11 (73%)	1 (7%)	3 (20%)	5	44
Adult outcome	1	1	1 (100%)	–	–	5	12	12 (100%)	–	–	5	13
**Health care**	**3**	**11**	**11 (100%)**	**–**	**–**	**2**	**10**	**7 (70%)**	**–**	**3 (30%)**	**5**	**21**
Pre‐teen outcome	2	9	9 (100%)	–	–	∼	∼	∼	∼	∼	2	9
Teen outcome	0	0	–	–	–	2	10	7 (70%)	–	3 (30%)	2	10
Adult outcome	1	2	2 (100%)	–	–	0	0	–	–	–	1	2
**Education**	**1**	**1**	**1 (100%)**	**–**	**–**	**4**	**32**	**26 (81%)**	**1 (3%)**	**5 (16%)**	**4**	**33**
Pre‐teen outcome	0	0	–	–	–	∼	∼	∼	∼	∼	0	0
Teen outcome	0	0	–	–	–	2	17	11 (65%)	1 (6%)	5 (29%)	2	17
Adult outcome	1	1	1 (100%)	–	–	2	15	15 (100%)	–	–	2	16
**Employment**	**1**	**2**	–	–	**2 (100%)**	**2**	**6**	**6 (100%)**	–	–	**2**	**8**
Pre‐teen outcome	0	0	–	–	–	∼	∼	∼	∼	∼	0	0
Teen outcome	0	0	–	–	–	0	0	–	–	–	0	0
Adult outcome	1	2	–	–	2 (100%)	2	6	6 (100%)	–	–	2	8
**Finance**	**1**	**1**	**–**	**–**	**1 (100%)**	**2**	**5**	**4 (80%)**	–	**1 (20%)**	**2**	**6**
Pre‐teen outcome	0	0	–	–	–	∼	∼	∼	∼	∼	0	0
Teen outcome	0	0	–	–	–	0	0	–	–	–	0	0
Adult outcome	1	1	–	–	1 (100%)	2	5	4 (80%)	–	1 (20%)	2	6
**Criminal justice**	**0**	**0**	**–**	**–**	**–**	**1**	**4**	**3 (75%)**	**–**	**1 (25%)**	**1**	**4**
Pre‐teen outcome	0	0	–	–	–	∼	∼	∼	∼	∼	0	0
Teen outcome	0	0	–	–	–	0	0	–	–	–	0	0
Adult outcome	0	0	–	–	–	1	4	3 (75%)	‐	1 (25%)	1	4
**Miscellaneous**	**1**	**3**	**–**	**–**	**3 (100%)**	**8**	**24**	**18 (75%)**	**1 (4%)**	**5 (21%)**	**8**	**27**
Pre‐teen outcome	0	0	–	–	–	∼	∼	∼	∼	∼	0	0
Teen outcome	0	0	–	–	–	4	11	6 (55%)	1 (9%)	4 (36%)	4	11
Adult outcome	1	3	–	–	3 (100%)	4	13	12 (92%)	–	1 (8%)	4	16

*Note*: Bold values represent overall results by exposure age.

The table summarises the direction of the effect between childhood anxiety problems and each outcome domain by age at exposure and age at outcome. NOTE: 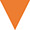 = outcome worsened; 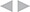 = no change in outcome (e.g. OR (odd ratio) = 1); 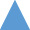 = outcome improved; Studies = number of studies; Analyses = number of analyses; – = no reported association; ∼ = not applicable.

Secondly, an effect direction plot was created to present the results of each outcome domain within and across studies (Boon & Thomson, 2021; McKenzie & Brennan, 2022). For each study (within‐study dimension), we counted whether exposure to anxiety problems improved or worsened the outcome in each analysis, and then determined an overall effect direction within each domain, at the study‐level, as follows: (a) if ≥ 70% of outcomes within a study reported the same ‘effect direction’, we reported that effect for the domain; (b) otherwise we reported no change/conflicting findings (Table 2).

**TABLE 2:** Effect direction plot of associations between anxiety exposure and each outcome domain summarised by study (subscript denotes number of associations).

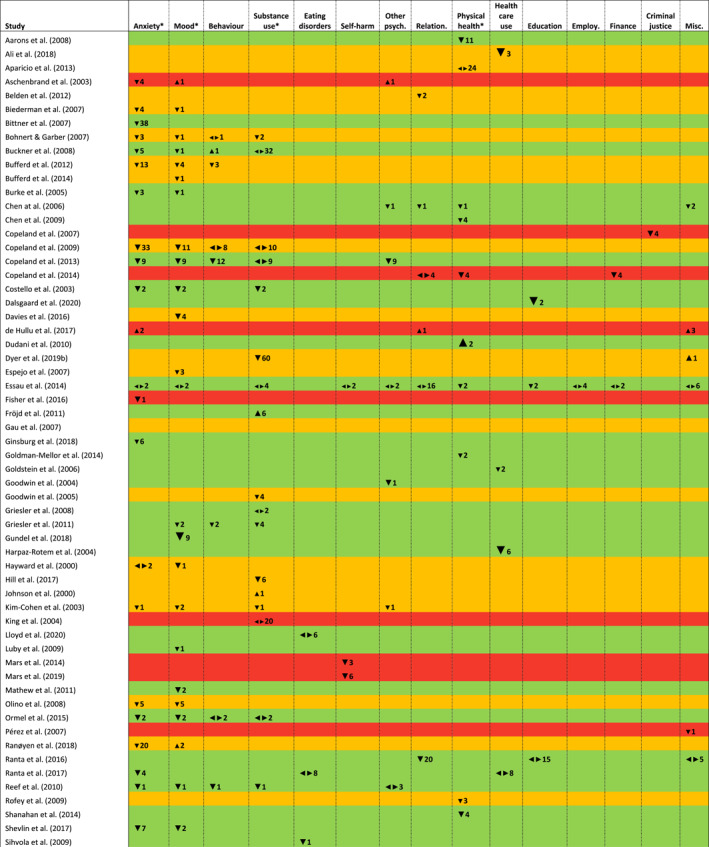
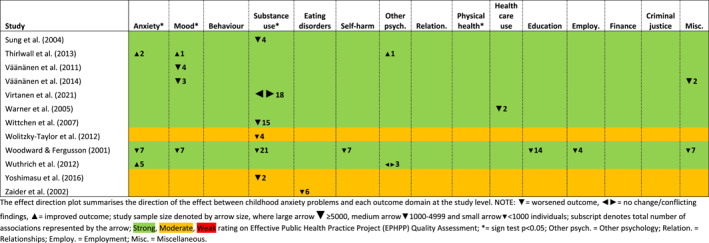

Finally, sign tests (Boon & Thomson, 2021; McKenzie & Brennan, 2022) were undertaken in Stata 17.0 (StataCorp, 2021), where possible, to test the null hypothesis of an equal number of worsened and improved associations across studies within each outcome domain (across‐study dimension). Study quality, based on EPHPP assessment, was incorporated in the plot through a traffic light system applied to each study.

##### Meta‐analysis

Secondly, meta‐analysis was undertaken whenever at least two longitudinal studies, from any given exposure‐outcome combination, met the following criteria (Appendix S4): exposure was a similar construct/measure; outcome was a similar construct/measure; same measure of effect reported (or calculated from study data); age at outcome, based on sample average age or upper age range limit if average not provided, fell within relevant age category, that is, childhood (≤18 years) or adulthood (≥19 years) (analyses spanning both age categories were included in the sensitivity analysis); and analysis was undertaken on different samples of individuals. Separate meta‐analyses were conducted for outcomes in childhood and adulthood to explore how outcomes differed by life stage. Meta‐analyses allowed us to quantify the magnitude of eligible associations across studies. Two or more studies are sufficient to conduct a meta‐analysis (Deeks et al., 2022; Ryan & Hill, 2016).

Random effects meta‐analysis using the DerSimonian‐Laird method was used to pool estimates of the association for each of the different exposure‐outcome combinations (DerSimonian & Laird, 1986). Heterogeneity across estimates was quantified using the I‐squared statistic, with the *p*‐value from Cochran's Q test used to assess evidence against homogeneity (Higgins et al., 2003). Publication bias was not assessed as none of the meta‐analyses included enough studies to detect meaningful evidence of bias (Sterne et al., 2011). Analysis was undertaken in Stata 17.0 (StataCorp, 2021) using the *metan* command (Harris et al., 2008).

##### Summary of results

Lastly, the overall results from the narrative synthesis and meta‐analyses were summarised graphically in Figure 2. Details on how to interpret Figure 2 are reported in detail in the associated legend. A *p*‐value of <0.05 was considered statistically significant throughout quantitative and narrative syntheses, as applicable.

#### Economic studies

The main results from all of the economic studies were synthesised narratively, with key characteristics, outcomes and costs of each study summarised in tabular form (Table 3). Among identified economic evaluations, we only included studies that reported the outcomes and costs of a waiting list, no intervention or treatment‐as‐usual control group. This is consistent with the approach undertaken with the longitudinal studies and our research aims, as we were interested in the outcomes and costs of child anxiety problems, rather than the cost‐effectiveness of given interventions.

**TABLE 3:** Descriptive analysis of key assessment items for included economic studies (*n* = 12).

Study	Country	Study design	Setting	Age range upon study entry	Sample size	Perspective	Time horizon	Economic outcome measures^1^	Cost type (Currency)^1^	Estimated economic outcomes (Control arm)	Estimated costs (2021 GBP) (Control arm)	EPHPP/CHEERS rating
D. H. Bodden, Dirksen, Bogels, et al. (2008)	Netherlands	Costing study	Health care	8–18 years with clinical anxiety	159	Societal	2 weeks extrapolated to 1 year	Not applicable	Direct and indirect (2003 EUR)	Not applicable	£3075 total societal per child; £1562 direct healthcare, £73 direct non‐healthcare, £1334 indirect costs, £107 OOP	Moderate/Not applicable
D. H. M. Bodden, Dirksen, Bogels, et al. (2008)	Netherlands	Trial‐based economic evaluation, CUA & CEA	‐	8–18 years with clinical anxiety	116	Societal	1 year follow‐up post‐treatment	QALYs (EQ‐5D), proportion children anxiety free on ADIS; 4% discount rate	Direct and indirect (2003 EUR); 4% discount rate	1.18 mean total QALYs per child, 68% of children anxiety free on ADIS at follow‐up (Treatment‐as‐usual)	£3079 total societal per child; £2147 direct healthcare, £58 direct non‐healthcare, £837 indirect costs, £37 out‐of‐pocket (Treatment‐as‐usual)	Moderate/95% (19 ÷ 20 = 0.95)
Chatterton et al. (2019)	Australia	Trial‐based economic evaluation, CUA	Health care	7–17 years (mean 9 years) with primary anxiety disorder	281	Societal	1 year follow‐up post‐treatment	QALYs (CHU‐9D, AQOL‐8D)	Direct and indirect (2015‐16 AUD)	0.69 and 0.79 mean total QALYs per child and parent respectively (Treatment‐as‐usual)	£2446 total societal per child; £1184 healthcare (Treatment‐as‐usual)	Moderate/95% (19 ÷ 20 = 0.95)
Costello et al. (2007)	USA	Costing study	Community	13–16 years with anxiety disorder or significant functional impairment due to symptoms	1420	Societal	3 months extrapolated to 1 year	Not applicable	Direct (2000 USD)	Not applicable	£3583 total direct societal per child, £965 for those with anxiety disorder only (i.e., no comorbidities)	Moderate/Not applicable
Creswell et al. (2017)	UK	Trial‐based economic evaluation, CUA & CEA	Health care	5–12 years (mean 9 years) with primary anxiety disorder	136	Societal	6 months follow‐up post‐treatment	QALYs (CHU‐9D, EQ‐5D‐Y), clinical improvement on CGI‐I of ‘much’ or ‘very much’	Direct and indirect (2013‐14 GBP)	0.88 and 0.91 utility score at baseline and follow‐up respectively, 72% clinical improvement on CGI‐I at follow‐up (Treatment‐as‐usual)	£2195 total societal per child (Treatment‐as‐usual)	Strong/100% (20 ÷ 20 = 1.00)
Creswell et al. (2020)	UK	Trial‐based economic evaluation, CUA	Community	7–12 years (mean 10 years) with anxiety disorder and a mother with anxiety disorder	211	Societal	1 year follow‐up post‐treatment	QALYs (EQ‐5D‐3L, EQ‐5D‐Y)	Direct and indirect (2011‐12 GBP)	1.67 mean total QALYs per child‐mother dyad (Treatment‐as‐usual)	£4679 total societal per child; £1279 NHS treatment, £343 child school absence, £521 mother missed work (Treatment‐as‐usual)	Strong/100% (20 ÷ 20 = 1.00)
Libutzki et al. (2019)	Germany	Costing study	Health care	0–17 years with inpatient or outpatient anxiety diagnosis	25,300	Societal	1 year	Not applicable	Direct (2014 EUR)	Not applicable	£1220 and £2180 total direct societal per child aged 0–12 and 13–17 years respectively	Moderate/Not applicable
Lim et al. (2016)	South Korea	Burden of disease study	Community	0–9 years with anxiety disorder	‐	Not applicable	1 year	DALYs, 3% discount rate	Not applicable	911 and 620 DALYs for males and females respectively	Not applicable	Moderate/Not applicable
Martin and Leslie (2003)	USA	Costing study	Community	0–17 years with anxiety disorder and engaged with mental health services	826,367	Healthcare provider	1 year	Not applicable	Direct (1997 USD)	Not applicable	£3610 total per child; £1881 and £1118 healthcare per inpatient and outpatient child respectively, £611 medication	Strong/Not applicable
Pella et al. (2020)	USA	Costing study	School	6–18 years (mean 10 years) with anxiety disorder	209	Societal	3 months extrapolated to 1 year	Not applicable	Direct and indirect (2016 USD)	Not applicable	£3809 total societal per child; £1479 direct costs, £2395 indirect costs	Moderate/Not applicable
Simon et al. (2012)	Netherlands	Trial‐based economic evaluation, CEA	School	8–12 years (mean 9 years) with high‐anxious screen for child anxiety related emotional Disorders‐71 (SCARED‐71) scores	139	Societal	2 years follow‐up post‐treatment	Proportion clinically improved on ADIS	Direct and indirect (2008 EUR); 4% discount rate	28% of children clinically improved on ADIS at follow‐up (No treatment)	£2580 total societal per child; £1855 direct healthcare, £106 direct non‐healthcare, £476 indirect, £142 OOP (No treatment)	Moderate/90% (18 ÷ 20 = 0.90)
van Steensel et al. (2013)	Netherlands	Costing study	Community	7–18 (mean 12 years) with at least 1 anxiety disorder	34	Societal	3 months	Not applicable	Direct and indirect (2010 EUR)	Not applicable	£4040 total societal per child; £1832 healthcare, £2210 non‐healthcare	Weak/Not applicable

Abbreviations: ‐, no information provided; 1, for studies with follow‐up longer than 1 year, discount rate is reported for both costs and health economic outcomes; ADIS, Anxiety and Related Disorders Interview Schedule; AQOL‐8D, Assessment of Quality of Life‐8 Dimentions; CBT, cognitive behavioural therapy; CEA, cost‐effectiveness analysis; CHU‐9D, Child Health Utility 9 Dimensions; CUA, cost‐utility analysis; DALY, disability‐adjusted life year; EQ‐5D‐3L, EuroQol‐5 Dimensions‐3 Levels; EQ‐5D‐Y, EuroQol‐5 Dimensions‐Youth; NHS, National Health Service; OOP, out‐of‐pocket.

All monetary values were converted into 2021 British pound sterling (GBP) prices, using a tool that utilises national price indices to calculate costs at a common date and International Monetary Fund purchasing power parities for currency conversion (Shemilt et al., 2010).

## RESULTS

**FIGURE 1:**
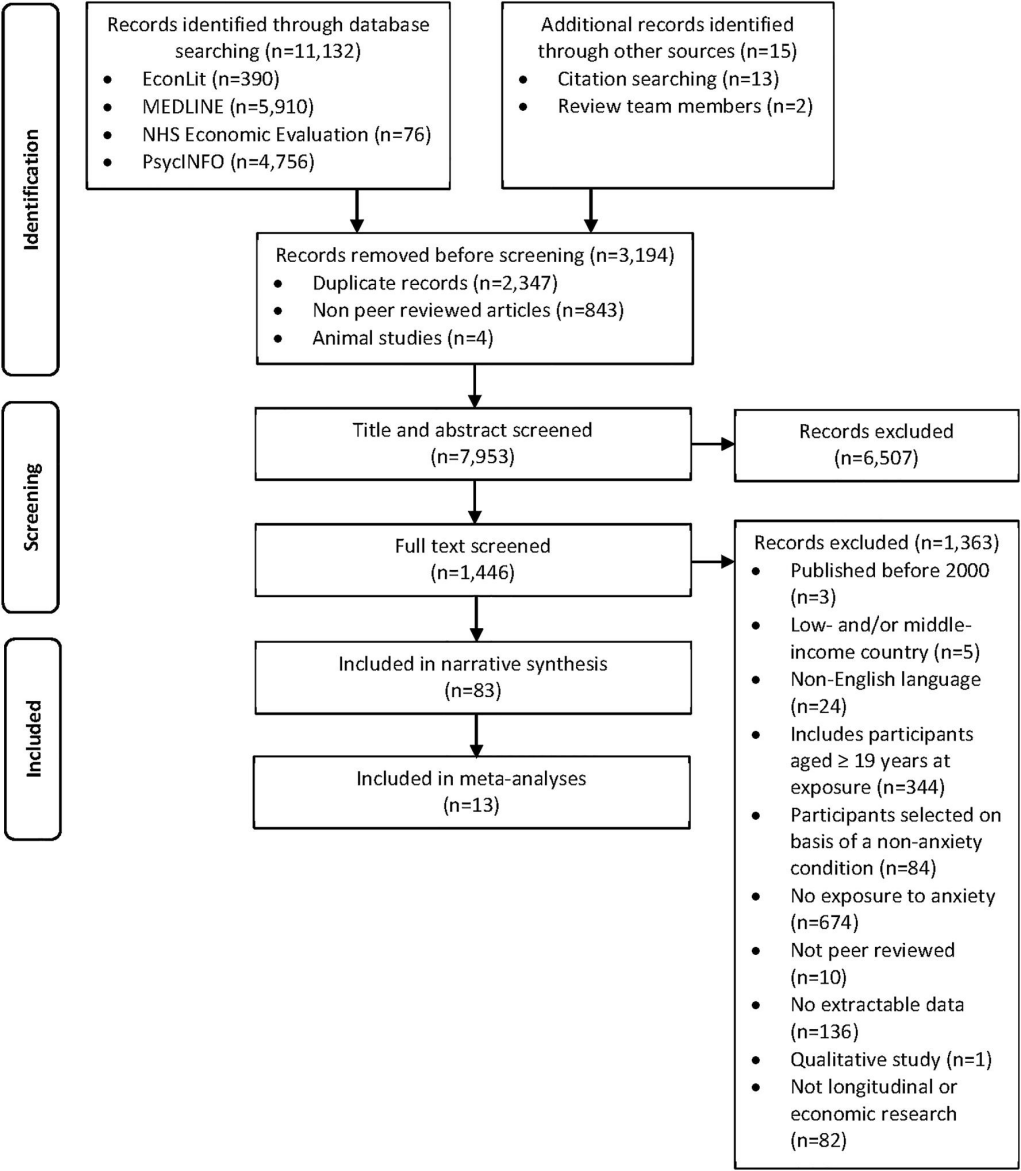
PRISMA flow diagram.

In total 7953 unique papers were identified through database searching and other sources, with 6507 excluded after title and abstract screening (Figure 1). Of the 1446 papers screened on full text, 83 were included in the narrative synthesis, with 13 of these included in the meta‐analyses. Seventy‐one of the 83 studies were longitudinal studies and 12 were economic studies.

### Longitudinal studies

#### Study characteristics

Characteristics of the longitudinal studies are summarised in Table S1 (Appendix S5) with full data extraction provided in Appendix S3.

#### Quality assessment

Table S1 (Appendix S5) includes the global rating of each paper on the EPHPP quality assessment tool. The majority of papers were judged to be of good quality, with 38 rated ‘Strong’, 23 ‘Moderate’ and 10 ‘Weak’. Common methodological weaknesses were failure to report the proportion of individuals agreeing to participate, incomplete or lack of adjustment for confounders, and high dropout rates or the failure to report them.

#### Data synthesis

**FIGURE 2:**
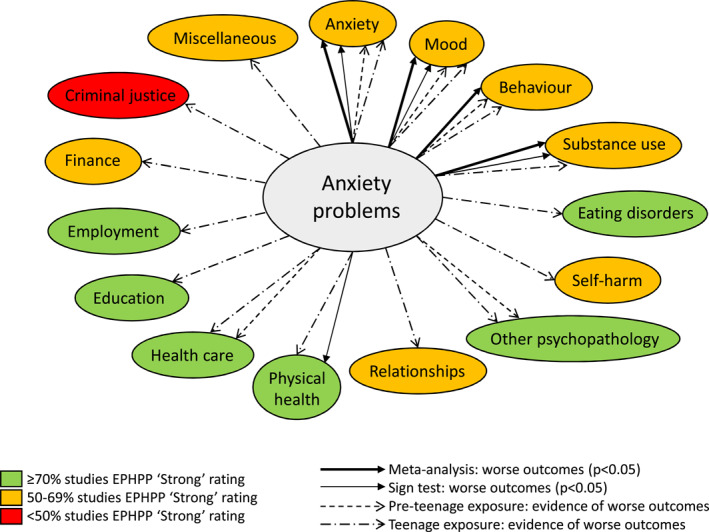
Strength and quality of evidence among longitudinal studies by outcome domain.

**FIGURE 3:**
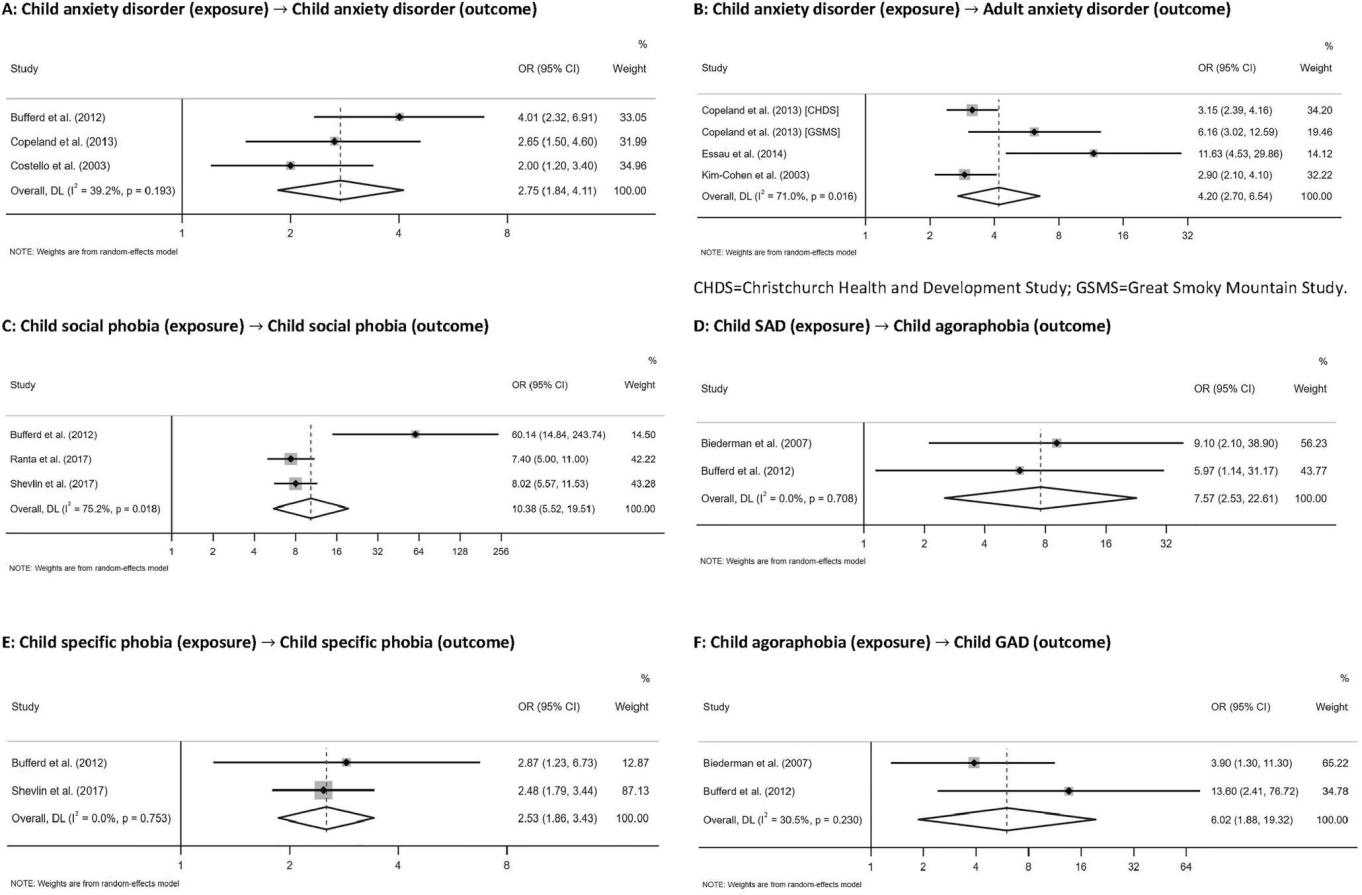
Meta‐analyses of the association between childhood anxiety and subsequent anxiety outcomes.

There were 788 associations in total across 71 studies; the direction of effect by exposure and outcome age are summarised in Table 1, and presented graphically in Figure S1 (Appendix S5). The effect direction plot in Table 2 summarises effect direction both within and across studies at the outcome domain level, recording statistically significant results based on the sign‐test, when relevant. Results from the meta‐analyses are shown in Figure 3 and Appendix S5, Figure S2 to S4, with sensitivity analyses in Appendix S6. Overall findings are summarised graphically in Figure 2. Follow‐up varied from 12 weeks to 27 years. Five studies had a follow‐up of less than a year, 33 had a follow‐up of 1 to less than 5 years, 31 had follow‐up of 5 years or more and two used a mixture of these follow‐up lengths. Only five studies had follow‐up beyond 20 years.

Figure 2 shows that children with anxiety problems were statistically significantly more likely to experience subsequent anxiety, mood, behaviour and substance use disorders, as per our meta‐analyses (Figure 3 and Appendix S5, Figure S2 to S4). Sign tests found an overall worse effect direction at the domain level for three of these four outcomes, that is, anxiety, mood and substance use outcomes, as well as for physical health outcomes (Figure 2 and Table 2). The narrative synthesis identified evidence of overall worse outcomes in each of the 15 outcome domains. Specifically, pre‐teenage and teenage anxiety problems were associated with worse subsequent anxiety, mood, behaviour, other psychopathology and healthcare related outcomes. All other domains were adversely affected by teenage anxiety problems, with no evidence of an association with pre‐teenage anxiety problems.

Study quality at the domain level, measured as more than 50% of included studies rated ‘strong’ on the EPHPP tool, was good in all but one of the outcome domains, criminal justice. A further summary of findings, organised by outcome domain, is presented below.

##### Anxiety outcomes

Twenty‐five studies, covering 181 associations (Table 1), were analysed, of which 20 reported an overall (at study level) worse effect direction, three improved and two were conflicting, with 14 (56%) being of strong quality (Table 2). Both pre‐teenage and teenage anxiety problems were associated with subsequent anxiety problems across all age groups (Table 1).

Pre‐teenage anxiety problems (exposure) were linked with worse subsequent pre‐teenage anxiety outcomes in 17 (89%) of the 19 analyses (Table 1), with higher frequencies of subsequent mixed anxiety disorders, agoraphobia, generalised anxiety disorder (GAD), panic disorder, selective mutism, separation anxiety disorder (SAD), social phobia and specific phobia (Appendix S3). Pre‐teenage anxiety problems (exposure) were also associated with poorer teenage anxiety outcomes in all 14 analyses, with teenage GAD, overanxious disorder, social phobia, and specific phobia all more likely to develop post‐exposure. Finally, 47 (80%) of 59 analyses reported worse anxiety outcomes in adulthood, with greater frequencies of agoraphobia, overanxious disorder, panic disorder, SAD and social phobia.

Similar results were identified in relation to teenage exposure to anxiety problems, which was associated with poorer subsequent teenage anxiety outcomes in 31 (82%) of 38 analyses (Table 1), with mixed anxiety disorders, agoraphobia, specific phobia, GAD, social phobia and pain related anxiety all more common after exposure. Adult anxiety outcomes were worsened in 44 (86%) of 51 analyses, with higher frequencies of mixed anxiety disorders, GAD, panic disorder, SAD and specific phobia.

##### Meta‐analysis

Anxiety disorders in childhood were significantly associated with subsequent anxiety disorders in childhood (OR = 2.75, 95% CI 1.84–4.11; *I* ^2^ = 39.2%; Figure 3A) and adulthood (OR = 4.20, 95% CI 2.70–6.54; *I* ^2^ = 71.0%; Figure 3B), although there was a high degree of heterogeneity between studies in the latter meta‐analysis. Considering specific anxiety disorders, social phobia in childhood was associated with later social phobia in childhood (OR = 10.38, 95% CI 5.52–19.51; *I* ^2^ = 75.2%; Figure 3C), although heterogeneity was high. Childhood SAD was associated with subsequent childhood agoraphobia (OR = 7.57, 95% CI 2.53–22.61; *I* ^2^ = 0%; Figure 3D). Childhood specific phobia was associated with later specific phobia in childhood (OR = 2.53, 95% CI 1.86–3.43; *I* ^2^ = 0%; Figure 3E), while childhood agoraphobia was associated with subsequent childhood GAD (OR = 6.02, 95% CI 1.88–19.32; *I* ^2^ = 30.5%; Figure 3F). All results remained consistent in the sensitivity analyses (Appendix S6).

##### Mood outcomes

Twenty‐eight studies, including 85 associations, were analysed (Table 1), of which 24 reported a worse effect direction, three improved and one was conflicting, with 16 (57%) being of strong quality (Table 2). Both pre‐teenage and teenage anxiety problems were associated with later mood problems across all age groups (Table 1).

Pre‐teenage anxiety problems were associated with worse later mood outcomes in pre‐teenagers in seven (88%) of eight analyses (Table 1), while all seven analyses considering teenage mood outcomes, and six (67%) of the nine analyses regarding adult mood outcomes reported poorer outcomes. Pre‐teenage anxiety problems were associated with subsequent pre‐teenage and teenage depression, and adult mixed mood disorders.

More evidence was found in relation to teenage anxiety problems, which were associated with poorer later teenage outcomes, such as depression, mood disorder and worse depression severity index scores, in 21 (91%) of 23 analyses (Table 1). Worse adult mood outcomes were also identified in 36 (95%) of 38 analyses, including depression and major depressive disorder.

##### Meta‐analysis

Anxiety disorders in childhood had a significant positive association with subsequent depression in childhood (OR = 2.16, 95% CI 1.52–3.08; *I* ^2^ = 0%; Figure S2A, Appendix S5) and adulthood (OR = 2.03, 95% CI 1.64–2.52; *I* ^2^ = 0%; Figure S2B, Appendix S5). However, the association between childhood anxiety disorders and subsequent childhood depression was no longer statistically significant in the sensitivity analysis, which included outcomes spanning both child and adulthood age categories, where the average age was less than 19 years (Appendix S6). Examining specific anxiety disorders, SAD in childhood had a significant positive association with depression later in childhood (OR = 4.05, 95% CI 1.84–8.91; *I* ^2^ = 0%; Figure S2C, Appendix S5).

##### Behaviour outcomes

From eight studies, covering 30 associations (Table 1), four reported an overall worse effect direction, one improved, and three were conflicting (Table 2).

Exposure to pre‐teenage anxiety problems (Table 1) was associated with subsequent oppositional defiant disorder (ODD) in pre‐teenagers, with all three analyses reporting worse outcomes, and teenage ODD, ADHD and conduct disorder, in all 12 analyses. Evidence on adult behaviour outcomes was mixed, with three (50%) of the six associations reporting poorer outcomes, two (33%) improved and one (17%) finding no change.

Teenage anxiety problems were associated with later teenage disruptive disorder in two (67%) of three analyses, while outcomes in adulthood were mixed, with four (67%) of the six associations reporting better behaviour outcomes, one (17%) worse outcome and one (17%) finding no change.

##### Meta‐analysis

Anxiety disorder in childhood had a significant positive association with subsequent ADHD in childhood (OR = 3.03, 95% CI 1.13–8.10; *I* ^2^ = 66.7%; Figure S3A, Appendix S5) and with subsequent ODD in childhood (OR = 2.30, 95% CI 1.47–3.60; *I* ^2^ = 0.0%; Figure S3B, Appendix S5).

##### Substance use outcomes

Considerable, if mixed, evidence was identified on the association between childhood problem anxiety and substance use, with 226 analyses across 24 studies (Table 1). Thirteen of the 24 studies reported an overall worse effect direction, three improved, and eight were conflicting (Table 2).

Pre‐teenage anxiety problems were associated with poorer teenage substance use outcomes in 23 (53%) of the 43 analyses, while 19 (44%) of the associations reported improved outcomes and one (2%) reported no change (Table 1). Similarly, six (60%) of the 10 associations between pre‐teenage anxiety and adult substance use reported worse outcomes, whereas the other four (40%) found improved outcomes.

Anxiety problems in teenagers were associated with worse subsequent teenage substance use outcomes in 12 (60%) of the 20 analyses. However, six (30%) associations found improved outcomes, while two (10%) reported no change. Adult substance use problems were reported in 113 (72%) of the 158 relevant analyses, and included SUD, alcohol dependence, alcohol use disorder, cannabis use, cannabis dependence, cannabis use disorder, nicotine dependence and illicit drug dependence.

##### Meta‐analysis

Anxiety disorder in childhood had a non‐significant positive association with subsequent SUD in childhood (OR = 1.57, 95% CI 0.94–2.63; *I* ^2^ = 23.4%; Figure S4A, Appendix S5), although the association was statistically significant in the sensitivity analysis (Appendix S6). Childhood anxiety disorder had a significant positive association with SUD in adulthood (OR = 1.26, 95% CI 1.05–1.52; *I* ^2^ = 37.8%; Figure S4B, Appendix S5).

##### Eating outcomes

Teenage anxiety problems were associated with worse eating outcomes in later teenage years, such as developing eating disorders and fasting for weight loss. Sixteen (76%) of 21 associations reported poorer outcomes (Table 1).

##### Self‐harm outcomes

Anxiety problems in teenagers were associated with poorer self‐harm outcomes in later teenage years, such as suicide ideation and attempts, as well as in adulthood, such as suicide attempts. All nine associations reported worse outcomes in teenagers, while all eight associations reported poorer outcomes in adulthood (Table 1).

##### Other psychopathology outcomes

Evidence was found of an association between childhood anxiety problems and worse outcomes in other psychopathology. Four (67%) of the six associations reported worse outcomes from pre‐teenage anxiety problems, such as developing any DSM disorder (Table 1). Teenage anxiety problems were associated with poorer outcomes in 12 (75%) of the 16 associations, with greater likelihood of psychosis, schizophreniform and chronic stress.

##### Relationship outcomes

Teenage anxiety problems were associated with poorer relationships, such as worse interpersonal functioning and perceived low relationship support, in twenty‐eight (82%) of 34 analyses (Table 1). However, eight (80%) of the 10 associations between pre‐teenage anxiety and subsequent relationship outcomes found improved outcomes, and the other two (20%) found worse outcomes.

##### Physical health outcomes

Anxiety problems in teenagers were associated with poorer physical health, such as lower health functioning, worse self‐reported physical health and more sleep problems, in 23 (85%) of 27 analyses (Table 1). Evidence on the association between pre‐teenage anxiety and physical health was mixed.

##### Health care outcomes

Anxiety problems in pre‐teenagers were associated with greater likelihood of treatment dropout, higher levels of unmet healthcare needs and increased health care use, such as higher medicine and therapy use, compared with their healthy counterparts, in all 11 analyses (Table 1). Eight (80%) of the 10 analyses considering teenage anxiety problems reported poorer outcomes, such as not seeking treatment for mental health problems.

##### Education outcomes

Evidence existed of an association between teenage anxiety problems and poorer education outcomes, with 26 (81%) of 32 analyses reporting worse outcomes, such as higher school absence, lower school completion and worse educational attainment (Table 1).

##### Employment outcomes

Teenage anxiety problems were associated with worse employment outcomes, although the evidence base was sparse. All six analyses reported poorer employment outcomes, such as greater likelihood of unemployment and lower ability to function in the workplace (Table 1). The two analyses considering pre‐teenage exposure reported improved employment outcomes.

##### Financial outcomes

There was some evidence of an association between teenage anxiety problems and poorer adult financial outcomes, although the evidence base was sparse. Four (80%) of the five analyses reported worse outcomes, such as lower household income and poorer financial functioning (Table 1). One analysis reported pre‐teenage anxiety problems were associated with improved financial outcomes.

##### Criminal justice outcomes

Some evidence of an association between teenage anxiety problems and adult criminal offences was identified. However, it was based on a single weak quality study. Three (75%) of the four analyses reported a greater likelihood of a criminal offence, with the other reporting a lower likelihood (Table 1).

##### Miscellaneous outcomes

Eighteen (75%) of the 24 analyses exploring the association between teenage anxiety problems and all other outcomes (i.e., miscellaneous) reported worse outcomes (Table 1), including lower self‐esteem in later teenage years, as well as poorer life satisfaction and coping skills in adulthood. All three analyses considering pre‐teenage anxiety problem exposure reported improved outcomes.

### Economic studies

#### Study characteristics

Characteristics of the economic studies are summarised in Table 3, with full data extraction provided in Appendix S3.

#### Quality assessment

Table 3 includes the global rating of each paper on the EPHPP quality assessment tool. Eight papers were judged to be of ‘Moderate’ quality, with three rated ‘Strong’ and one ‘Weak’. Table 3 also includes the overall CHEERS score of the trial‐based economic evaluations, with one paper meeting 90% (18/20) of the checklist, two 95% (19/20) and two 100% (20/20).

#### Data synthesis

Table 3 reports detailed estimated economic outcomes and costs from each of the 12 included studies.

Six were costing studies, four of which took a societal perspective to estimate the cost‐of‐illness of anxiety disorder using a prevalence‐based approach. Among these (Bodden, Dirksen, & Bogels, 2008; Libutzki et al., 2019; Pella et al., 2020; van Steensel et al., 2013), estimated total annual costs varied from £1220 per German child aged 0–12 years (Libutzki et al., 2019) to £4040 per Dutch child mean aged 12 years (van Steensel et al., 2013), although indirect costs were not considered in the former study. The two other costing studies were US based. Costello et al. (2007) estimated total annual direct costs of children aged 13–16 with anxiety disorders to be £3,583, although this dropped to £965 when excluding children with other comorbidities. Martin and Leslie (2003) found that the annual direct costs of privately insured children aged 0–17 years with anxiety disorders totalled £3610.

One study estimated the burden of disease in South Korean children aged 0–9 years in 2012, reporting that anxiety disorder resulted in 911 and 620 disability‐adjusted life years for males and females respectively (Lim et al., 2016).

Five studies undertook trial‐based economic evaluations of interventions treating children with anxiety problems. Creswell et al. (2017) reported mean total societal costs of £2195 per UK child mean aged 9 in the control arm, with direct and indirect costs considered across 6 months of follow‐up. Child Health Utility 9D child‐report (CHU‐9D‐c), which is a paediatric generic preference‐based measure of health‐related quality of life (HRQoL) ranging from 0 (death) to 1 (full health), varied from 0.88 (baseline) to 0.91 (follow‐up) in the control arm. In another UK study, Creswell et al. (2020) found mean total direct and indirect societal costs and QALYs gained (child and mother combined) in the control arm for the child‐mother dyad were £4679 and 1.67 respectively, over the one‐year follow up. Bodden, Dirksen, Bogels, et al. (2008) estimated mean total societal costs of £3079 per Dutch child mean aged 12 in the control arm, considering direct and indirect costs across the treatment period and 1‐year follow‐up post‐treatment, with a mean 1.18 QALYs reported. In an Australian study of children mean aged 9, Chatterton et al. (2019) reported direct and indirect mean total societal costs of £2446 per child in the control arm after one‐year follow‐up, with mean total QALYs being 0.693 and 0.792 for the children and the parents, respectively. Finally, after two years' follow‐up of Dutch children mean aged 9, Simon et al. (2012) found mean total direct and indirect societal costs of £2580 per child.

## DISCUSSION

This review synthesised 83 studies to provide a comprehensive overview of the multiple outcomes of anxiety problems in childhood, and the associated costs. Seventy‐one longitudinal studies, accounting for 788 separate analyses, were synthesised narratively, using effect direction as a common metric and summarised by an effect direction plot, after grouping outcomes into 15 overarching domains. Thirteen meta‐analyses were also undertaken, pooling estimates from 13 of the studies, covering anxiety, mood, behaviour, and SUD outcomes. Worsened effect direction at the domain level was found for anxiety, mood, substance use, and physical health outcomes, based on results of the sign test. For the other domains, there was insufficient evidence to establish an overall effect direction, but most effect directions at the study level pointed towards worsening outcomes, with some reporting overall conflicting directions. Twelve economic studies were also narratively synthesised, covering trial‐based economic evaluations, costing studies, and one burden of disease study. We found that childhood anxiety problems were associated with negative and enduring clinical and economic outcomes in later childhood, adolescence, and early adulthood, as well as higher economic costs compared with not having anxiety problems, especially when comorbidities were present. Overall, most (87%) of the identified studies were of strong (41) or moderate (31) quality, which suggests that the findings presented have a reasonable degree of reliability.

We identified clear evidence of a significant association between childhood anxiety problems and subsequent anxiety and mood problems, regardless of age at exposure or outcome. Although the natural history of anxiety disorders across the lifespan is often heterogeneous, previous evidence suggests that 40%–60% of anxiety disorders develop a persistent course (Hovenkamp‐Hermelink et al., 2021). In light of this, our results underscore the importance of developing interventions aimed at prevention or early identification and treatment of anxiety problems in children, with the aim of improving childhood HRQoL and avoiding chronic anxiety disorders, and associated mood disorders, over the life course. Indeed, our findings found a clear association between childhood anxiety disorders and subsequent mood disorders, consistent with other published reviews (Cerda et al., 2008; Schleider et al., 2014).

A considerable amount of evidence indicated an overall worsened effect direction between childhood anxiety problems and adult substance use outcomes, but evidence was mixed when considering earlier age outcomes. Other reviews drew similar conclusions (Dyer et al., 2019; Groenman et al., 2017; Ning et al., 2020). This may be due to the heterogeneity of anxiety disorders and the typical age at onsets. For example, those with a social anxiety disorder, which typically onsets by age 13 (Solmi et al., 2021), may be at lower risk as they have less exposure to peers who may use substances. However, anxiety disorders with a later median onset age, like GAD, may have a higher risk for later substance use (Fröjd et al., 2011).

We found clear evidence of an association between anxiety problems and worsened behaviour outcomes, mainly among pre‐teenagers and teenagers, with mixed results in adulthood. Results of other reviews were consistent with ours (Cerda et al., 2008; D'Agati et al., 2019). This may be a result of overlapping anxiety and behaviour problem symptoms in children, which may lead to the diagnosis of both, when in fact only one is present (D'Agati et al., 2019), or a consequence of the development course of behaviour problems being intertwined with internalising problems during adolescence (Measelle et al., 2006).

Previous studies have documented the co‐occurrence of anxiety problems and poorer physical health among children and adolescents (Balázs et al., 2018; Chavira et al., 2008). In our review, however, evidence was mixed when considering pre‐teenage exposure to anxiety problems, but associations with worsened physical health appeared stronger when considering teenage exposure and adult outcomes. This may be a result of physical health problems being more likely to develop with age and/or their cumulative health burden increasing over time when chronic, while only one study in our review explored the association between pre‐teenage anxiety problems and adult physical health outcomes (Essau et al., 2014).

For the other remaining 10 outcome domains identified within the longitudinal studies (Figure 2), the sign test could not establish an effect direction at domain level, although some evidence of worsening outcomes emerged consistently, at study level, mainly in relation to teenage exposure. Interestingly, four of these 10 domains were about health or health‐related outcomes (self‐harm, eating disorder, other psychopathology, health care use), bringing the total number of health‐pertaining domains to nine (60%) out of our 15 outcome domains. Our findings on self‐harm outcomes did not confirm the findings of a previous a meta‐analysis of 20 studies, which found that anxiety disorders were not significantly associated with suicide attempt (Gili et al., 2019). However, this may be a result of the breadth of outcomes included in our self‐harm outcome domain, which included self‐harm without suicidal intent and suicidal ideation without attempt. The association identified between teenage anxiety problems and adverse eating outcomes in later teenage years replicates another review, which reported that all anxiety disorder diagnoses combined predicted an increased risk of anorexia nervosa (Lloyd et al., 2019).

Three (30%) out of the 10 domains (education, employment, and finance outcomes) often adversely affected by childhood anxiety disorders, but for which meta‐analysis was not feasible (Figure 2), represent a substantial impact on function and development throughout life, with long term negative consequences at the individual, family and societal level identified. Yet, only five studies (7%) out of 71 included these important economic aspects. This may be due to a lack of longitudinal studies that measure anxiety problems rigorously and have sufficient follow‐up, from childhood to adulthood, to measure such outcomes. In fact, all five studies considered teenage exposure to anxiety problems, with poorer outcomes identified in final year exam grades, entering tertiary education or training, and academic progress. Similarly, Finning et al. (2019) found anxiety problems were associated with worse education outcomes in their review, while Riglin et al. (2014) reported anxiety problems had a positive association with school failure, although not with school grades. We found little evidence on the longitudinal association between anxiety problems and employment and finance outcomes. The study exploring pre‐teenage anxiety exposure gave inconsistent results, pointing to improved employment and finance outcomes, but worsened education outcomes. Meanwhile, the sparse evidence on financial outcomes consistently pointed to worse financial outcomes in adulthood, following teenage anxiety problems. Future studies investigating and quantifying the impact of childhood anxiety problems on educational and labour market outcomes are therefore warranted, in order to better understand the overall burden of anxiety disorders beyond health‐related domains.

Our review also revealed adverse impacts on social relationships in teenage years, corroborating previous studies showing that anxiety in adolescence was associated with poor peer relationships and friendships (De Matos et al., 2003; La Greca & Lopez, 1998). Sparse evidence on criminal outcomes existed, with limited evidence identified of an association between teenage anxiety problems and adult criminal offences based on a single weak quality study. Similarly, a previous review found that childhood internalising issues, including depression and anxiety, were a modest predictor of adult criminality, with behavioural issues playing a much greater role (Leschied et al., 2008). This suggests that caution is needed when interpreting potential associations between childhood anxiety problems and criminal outcomes, as they may in fact reflect comorbidities or other unobserved confounders.

On the whole, not only did the longitudinal studies mainly focus on health‐related outcomes, with most evidence pointing towards a negative impact of childhood anxiety problems, they also primarily investigated consequences at the individual child‐level, without considering the wider impacts on families and wider society. This may be a result of the limitations of the available data, which mainly followed children for limited periods of time and did not capture information on the potential burden of childhood anxiety beyond the children themselves. Yet family‐ and societal‐level perspectives are important when assessing the overarching consequences of child mental health problems and crucial when making resource allocation decisions, and future research should explore outcomes in these groups.

The identified economic studies indicated that total average direct and indirect societal costs were up to £4040 annually per clinically anxious child (van Steensel et al., 2013). These costs are lower than those associated with autism spectrum disorder, with equivalent costs amounting to £8110 annually in young children (Buescher et al., 2014). However, comparable annual costs associated with subclinical and clinical depression were £705 and £1960 respectively (Denise HM Bodden, van den Heuvel, Engels, & Dirksen, 2022), considerably lower than those associated with clinically anxious children.

The paucity of economic outcomes found in the included longitudinal studies was mirrored by the scarcity of specific economic studies identified in our review. This is clearly symptomatic of a more systematic lack of economic evidence in the anxiety disorders literature. For instance, a previous review of economic studies only identified one cost‐of‐illness article for anxiety, with more studies for autistic spectrum disorder, ADHD and conduct disorder (Beecham, 2014). Similarly, in their review of economic evaluations of youth mental health care interventions (excluding pharmacological or individual psychological therapies for full threshold disorders), Hamilton et al. (2017) only identified two studies focussed specifically on anxiety disorders. Among the studies identified in our review, anxiety problems were associated with considerable direct (e.g. health care) and indirect costs (e.g. informal care), and affected not only the individual child (e.g. missed school), but also their family (e.g. parent time off work) and wider society (e.g. lost productivity). The importance of documenting child mental health ‘spillover effects’ on the family and wider society, in terms of both health outcomes and costs, has been much emphasised, especially in the economic literature (Al‐Janabi et al., 2016; Knapp & Wong, 2020; Tubeuf & Guthmuller, 2017), yet evidence on these aspects is still scarce. Future research should therefore address these important gaps in knowledge both in economic studies as well as more general longitudinal studies, as previously emphasised.

Our review also highlighted the short time horizons of all the economic studies considered, with the longest follow up period being 2 years. As demonstrated, childhood anxiety problems have pervasive and long‐lasting clinical and economic impacts, so another important direction for future research is to investigate the long‐term economic consequences of these debilitating illnesses (Knapp & Wong, 2020; Tubeuf & Guthmuller, 2017).

The major strength, and originality, of our review is its comprehensiveness, without compromising on quality or rigour, following best practice guidelines for undertaking (Garritty et al., 2021) and reporting rapid systematic reviews (Moher et al., 2009). Accounting for the complex consequences of childhood anxiety problems, we have covered outcomes affecting multiple, individual‐level, domains of daily life (health, education, employment, social relations), but also captured ‘spillover effects’ on families and wider society (health care, education, and labour market engagement). Furthermore, stratification of results based both on age at exposure and outcome, gives an overview of consequences over the life span. Following from this, our extremely detailed and interactive extraction form (Appendix S3) is a useful tool for a multiplicity of stakeholders, from clinicians and practitioners to health economists and policy‐makers, as results can be filtered by characteristics of interest, for a quick yet comprehensive overview. Use of economic models that predict future outcomes should be encouraged whenever longitudinal studies with sufficient follow‐up are not available, and our extraction form will facilitate those wishing to identify economic model inputs specific to childhood anxiety outcomes, as the effect size and relevant uncertainty of numerous exposure‐outcome combinations are reported in detail, complete with economic costs, when applicable.

The results of our systematic review should also be interpreted in light of some limitations. The variety of identified outcomes, captured in 15 overarching mutually exclusive domains, made synthesising the longitudinal studies challenging. Ideally, we would have conducted meta‐analyses to quantitatively combine data across all studies. However, the aforementioned variety of outcomes meant this was not feasible. Furthermore, the considerable number of associations identified meant we were unable to summarise their individual effect sizes and relevant uncertainty in the main article text. Therefore, to enable overall synthesis, we categorised each association based on the direction of its effect (Table 1), generated an effect direction plot, and undertook sign tests where possible (Table 2). Our approach does not provide an overall estimate of the effect size of the association for given exposure‐outcome combinations, which is needed to assess clinical importance. As a result, the effect direction plot may overestimate effect direction in some circumstances and underestimate it in others. Moreover, the method does not summarise the imprecision (e.g., using confidence intervals) of the estimates so it is not possible to convey the extent to which a larger sample (or more studies) would be needed to obtain definitive conclusions. Furthermore, the power of the sign test at domain level may be limited where the outcome domain contains few studies, especially if some report conflicting findings, as these are excluded from the sign test (Boon & Thomson, 2021). Therefore, the summary reported in Table 1 needs to be considered in light of these limitations and interpreted in conjunction with the full information reported in the extraction form (Appendix S3). However, it is important to note that our approach was in line with the guidance in the 2022 Cochrane Handbook (McKenzie & Brennan, 2022).

The largest meta‐analysis that we conducted only included four studies. Consequently, effect estimates were less certain and the representation of between‐study variation was limited (Borenstein et al., 2021). We were also unable to assess publication bias as there were too few studies to detect meaningful evidence of bias (Sterne et al., 2011). However, such bias may have been present. Furthermore, the relationships we identified throughout the review were mainly associational. Despite our focus on consequences measured after exposure to anxiety problems, which partially addressed concerns of reverse causality, we could not exclude problems linked to unobserved heterogeneity, unless the paper reviewed explicitly set out to establish causal relationships. Finally, some studies focussed on specific groups of people, such as those in specific geographical regions or based in certain study settings, for example, hospital patients. Therefore, although such studies captured representative samples of the given context, and in some cases oversampled underrepresented individuals (e.g. ethnic minorities), the representativeness of their results for wider populations needs to be considered with caution. Despite these limitations, our review draws on well‐established methodologies to provide an overview of the multitude of consequences of childhood anxiety problems.

## CONCLUSION

The evidence base, that we have identified and summarised in this rigorous and comprehensive systematic review, overwhelmingly indicates that childhood anxiety problems are associated with a variety of worse outcomes. There was also some evidence of associations with improved outcomes in certain contexts, but the evidence base was not strong enough to draw any conclusive interpretation. Where feasible, we provided potential explanations of such improved outcomes by drawing on wider published evidence, and our transparent reporting will allow the interested reader to further explore such associations. Our review provides a holistic overview of the consequences of child anxiety problems, supplying important evidence for much needed future research on modelling the long‐term economic outcomes, to inform the allocation of resources to address the detrimental clinical and economic impacts of child anxiety problems. Our findings suggest that child anxiety problems persist in later childhood, teenage and early adult years, and are associated with various enduring adverse outcomes, including mood, behaviour, substance use and behaviour disorders, as well as worse self‐harm, educational, physical health and health care outcomes, while further research is required on employment, finance and criminal justice outcomes. Moreover, child anxiety problems were associated with substantial child‐, family‐ and societal‐level economic costs, all of which highlights the urgent need for cost‐effective preventative and interventional policies to address the issue.

## AUTHOR CONTRIBUTION

**Jack Pollard**: Conceptualization, Data curation, Formal analysis, Investigation, Methodology, Visualization, Writing – original draft, Writing – review & editing. **Tessa Reardon**: Conceptualization, Methodology, Writing – review & editing. **Chloe Williams**: Conceptualization, Investigation, Writing – review & editing. **Cathy Creswell**: Conceptualization, Funding acquisition, Methodology, Writing – review & editing. **Tamsin Ford**: Conceptualization, Funding acquisition, Writing – review & editing. **Alastair Gray**: Conceptualization, Funding acquisition, Writing – review & editing. **Nia Roberts**: Conceptualization, Resources, Writing – review & editing. **Paul Stallard**: Conceptualization, Funding acquisition, Writing – review & editing. **Obioha C. Ukoumunne**: Conceptualization, Funding acquisition, Methodology, Writing – review & editing. **Mara Violato**: Conceptualization, Funding acquisition, Investigation, Methodology, Project administration, Supervision, Writing – original draft, Writing – review & editing.

## CONFLICTS OF INTEREST STATEMENT

TF's team receives funding from Place2Be, a third sector organisation providing mental health support for schools that supports team members. The remaining authors have declared that they have no competing or potential conflicts of interest.

### OPEN RESEARCH BADGES

This article has earned a Preregistered Research Designs badge for having a preregistered research design, available at https://www.crd.york.ac.uk/prospero/display_record.php?ID=CRD42021202440.

## ETHICAL CONSIDERATIONS

Ethical approval was not required for this review article.

## Supporting information

Supplementary Material

Supplementary Material

Supplementary Material

## Data Availability

The data that supports the findings of this study are available in the supplementary material of this article.
