# Supplementary material for: The multifaceted consequences and economic costs of child anxiety problems: A systematic review and meta‐analysis
Source: JCPP Adv. 2023 Apr 21;3(3):e12149. doi: 10.1002/jcv2.12149 (PMC10501703; doi:10.1002/jcv2.12149)

**Supporting Information**

**Appendix S1. Literature review inclusion and exclusion criteria**

| *Category* | *Inclusion criteria* | *Exclusion criteria* |
| --- | --- | --- |
| Publication date | Studies published from 2000. | Studies published before 2000. |
| Geography | Research about high-income countries, as defined by the World Bank.^1^ | Research about low- and middle-income countries, as defined by the World Bank.^1^ |
| Language | English. | All other languages. |
| Population | Children and young people younger than 19 years at time of exposure. | Studies where some or all participants are aged 19 years or over at time of exposure.  Studies examining individuals selected specifically on the basis of a non-anxiety mental, physical or neurodevelopmental condition. |
| Exposure | Presence of an anxiety disorder or elevated anxiety symptoms on the basis of (i) a DSM-5 or ICD-10 code, (ii) a diagnosis using a gold standard semi-structured interview (e.g. Anxiety Disorders Interview Schedule [ADIS]) or from a trained professional, or (iii) an established cut-off score on a validated self-, parent/carer- or teacher-reported questionnaire measure. | Study **does not** report the presence of an anxiety disorder or elevated anxiety symptoms. |
| Study type | Peer reviewed articles. | Non-peer reviewed articles. |
|  | Quantitative studies that provide extractable data on the direct association between childhood anxiety disorders or elevated anxiety symptoms and at least one individual-, family- or societal-level outcome. | Studies that **do not** provide extractable data on the direct association between childhood anxiety disorders or elevated anxiety symptoms and at least one individual-, family- or societal-level outcome.  Studies using qualitative methods only. |
|  | Research that is one or more of the following:   - Longitudinal study; - Cohort study with follow-up; - Economic study; cost of illness, economic evaluation (cost-effectiveness, cost-utility, cost-benefit, cost-minimisation analysis, economic evaluation alongside randomised controlled trials).^2^ | All other research. |
| Study participants | Human participants, younger than 19 years at the time of exposure. | Non-human participants and human participants aged 19 years and above at the time of exposure. |
| Outcomes | Impact of child and young person anxiety disorders or elevated anxiety symptoms on at least one individual-, family- or societal level-outcome. | All other outcomes. |
|  | Costs associated with the impact of child and young person anxiety disorders or elevated anxiety symptoms on at least one individual-, family- or societal-level outcome. | All other costs. |

^1^ The World Bank classifies countries into four income groups (low, lower-middle, upper-middle, and high) based on the World Bank Atlas gross national income per capital estimates. For more information see: <https://datahelpdesk.worldbank.org/knowledgebase/articles/906519-world-bank-country-and-lending-groups> (Accessed 1 July 2022).

^2^ Amongst identified economic evaluations, we only included studies that reported the outcomes and costs of a waiting list, no intervention or treatment-as-usual control group.

**Appendix S4. Meta-analysis inclusion criteria**

At least two studies from any given exposure-outcome combination, for childhood or adulthood outcomes, had to meet the following selection criteria in order for a meta-analysis to be undertaken:

- Exposure of interest is a similar construct and measure across studies (e.g. anxiety disorder based on a validated measure).
- Outcome of interest is a similar construct and measure across studies (e.g. depressive disorder based on a validated measure).
- Measure of effect is the same across studies (e.g. odds ratio).
  - If the reported measure of effect differed, we checked the study for raw data and where possible calculated the measure of effect of interest in order to include it in the meta-analysis.
- Age at outcome falls within our relevant age category definition (e.g. childhood <19 years, adulthood ≥19 years).
  - If the age at outcome spanned both age category definitions (e.g. 16-21 years), the study was not included in the main meta-analysis, but was included in a sensitivity analysis. To determine age at outcome and therefore which sensitivity analysis to include it in, childhood or adulthood, we used the sample average age, or the mid-point of the age band if sample average age was not reported (e.g. 18.5 years in the example above).
- Studies included in a given meta-analysis do not have overlapping samples of individuals.
- Where more than one analysis (across all studies) met all of the above criteria for a given exposure-outcome combination, we included the analysis which controlled for most variables.
- Where a given study included more than one analysis that met all of the above criteria for a given exposure-outcome combination, we included the analysis which was most similar in age at exposure to the other studies in the meta-analysis.
  - If age at exposure was the same across multiple analyses within a study, we selected only the analysis with the latest age at outcome.

**Appendix S5. Supplementary tables and figures**

Table S1: Characteristics of included longitudinal studies (n=71)

| **Study** | **Country** | **Study design** | **Setting** | **Sample size^1^** | **Gender^2^** | **Exposures** | **Exposure measurement type** | **Age at exposure^3^** | **Outcomes** | **Age at outcome^3^** | **EPHPP Global Rating** |
| --- | --- | --- | --- | --- | --- | --- | --- | --- | --- | --- | --- |
| Aarons et al. (2008) | USA | Register-based study | Community | 1,332 | Female, male (proportions not specified) | 1) Anxiety disorder | Diagnostic assessment | 9-18 years (mean [SD] 14 [2.4] years) | 1) Health problems  2) Infectious diseases  3) Respiratory issues  4) Risky health-related behaviour  5) Weight problems | 11-20 years | Strong |
| Ali et al. (2018) | USA | Register-based study | Community | 1,987,759 | 49% female, 51% male | 1) Anxiety disorder | Medical records | 0-5 years | 1) Psychotropic prescription  2) Psychotherapy treatment | Up to 1 year after exposure | Moderate |
| Aparicio et al. (2013) | Spain | Survey-based longitudinal study | School | 142 in female analysis, 87 in male analysis | Female, male | 1) Separation anxiety disorder  2) Generalised anxiety disorder  3) Panic disorder  4) Social phobia | Diagnostic assessment | 10 [0.9] years (mean [SD]) | 1) Waist circumference  2) Body mass index  3) Percent body fat | 13 [0.9] years (mean [SD]) | Moderate |
| Aschenbrand et al. (2003) | - | Survey-based longitudinal study | Health care | 85 | 36% female, 64% male | 1) Separation anxiety disorder | Diagnostic assessment | 9-13 years | 1) Comorbid panic disorder, agoraphobia and major depressive disorder  2) Generalised anxiety disorder  3) Panic disorder  4) Social phobia  5) Other anxiety disorders  6) Major depressive disorder | 15-22 years (mean [SD] 19 [1.7] years) | Weak |
| Belden et al. (2012) | - | Survey-based longitudinal study | Community | 121 | 52% female, 48% male | 1) Anxiety disorder | Diagnostic assessment | 3-5 years | 1) Relational aggression perpetrators/victims | 2 years after exposure | Moderate |
| Biederman et al. (2007) | USA | Survey-based longitudinal study | Community | 215 | 47% female, 53% male | 1) Separation anxiety disorder  2) Agoraphobia | Medical records | 5-13 years (mean 6 years) | 1) Specific phobia  2) Agoraphobia  3) Panic disorder  4) Depression  5) Generalised anxiety disorder | 7-18 years (mean 10 years) | Moderate |
| Bittner et al. (2007) | USA | Survey-based longitudinal study | Community | 906 | 44% female, 56% male | 1) Generalised anxiety disorder  2) Overanxious disorder  3) Separation anxiety disorder  4) Social phobia | Diagnostic assessment | 9-12 years | 1) Agoraphobia  2) Generalised anxiety disorder  3) Overanxious disorder  4) Panic attack  5) Separation anxiety disorder  6) Social phobia  7) Specific phobia | 13-19 years | Strong |
| Bohnert and Garber (2007) | USA | Survey-based longitudinal study | School | 198 | 57% female, 43% male | 1) Anxiety disorder | Diagnostic assessment | 13-14 years | 1) Anxiety disorder  2) Mood disorder  3) Behaviour disorder  4) Substance use disorder  5) Tobacco use | 17-18 years | Moderate |
| Buckner et al. (2008) | USA | Survey-based longitudinal study | School | 816 | 54% female, 46% male | 1) Social anxiety disorder  2) Panic disorder  3) Overanxious disorder  4) Specific phobia  5) Separation anxiety disorder | Diagnostic assessment | 16 [1.2] years (mean [SD]) | 1) Alcohol abuse  2) Alcohol dependence  3) Cannabis abuse  4) Cannabis dependence  5) Separation anxiety disorder  6) Mood disorder  7) Conduct disorder  8) Panic disorder  9) Obsessive compulsive disorder  10) Generalised anxiety disorder  11) Specific phobia | 30 [0.6] years (mean [SD]) | Strong |
| Bufferd et al. (2012) | USA | Survey-based longitudinal study | Community | 462 | 46% female, 54% male | 1) Anxiety disorder  2) Agoraphobia  3) Social phobia  4) Specific phobia  5) Separation anxiety disorder  6) Selective mutism | Diagnostic assessment | 3 [0.3] years (mean [SD]) | 1) Anxiety disorder  2) Agoraphobia  3) Social phobia  4) Specific phobia  5) Separation anxiety disorder  6) Selective mutism  7) Generalised anxiety disorder  8) Depression  9) ADHD  10) Oppositional defiant disorder | 6 [0.4] years (mean [SD]) | Moderate |
| Bufferd et al. (2014) | USA | Survey-based longitudinal study | Community | 456 | 46% female, 54% male | 1) Anxiety disorder | Diagnostic assessment | 3 [0.3] years (mean [SD]) | 1) Depression | 6 [0.4] years (mean [SD]) | Moderate |
| Burke et al. (2005) | USA | Survey-based longitudinal study | Health care | 177 | 100% male | 1) Overanxious disorder | Diagnostic assessment | 7-12 years | 1) Overanxious disorder  2) Depression | 1 year after exposure, up to 18 years | Weak |
| Chen et al. (2006) | USA | Survey-based longitudinal study | Community | 608 | 55% female, 45% male | 1) Anxiety disorder | Diagnostic assessment | 16 (±2.8) years | 1) Young Adult Quality of Life (YAQOL) Physical Health Dimension  2) YAQOL Social Relationships Dimension  3) YAQOL Psychological Well-being Dimension  4) YAQOL Environmental Context Dimension | 33 (±2.8) years | Strong |
| Chen et al. (2009) | USA | Survey-based longitudinal study | Community | 564 | 50% female & 50% male | 1) Anxiety disorder | Diagnostic assessment | 13 years (mean) | 1) Pain  2) Physical illness  3) Physical health | 16-33 years | Strong |
| Copeland et al. (2007) | USA | Survey-based longitudinal study | Community | 1,420 | 44% female, 56% male | 1) Anxiety disorder | Diagnostic assessment | 9-16 years | 1) Criminal offences | 16-21 years | Weak |
| Copeland et al. (2009) | USA | Survey-based longitudinal study | Community | 1,420 | Female, male (proportions not specified) | 1) Overanxious disorder  2) Generalised anxiety disorder  3) Separation anxiety disorder | Diagnostic assessment | 13-16 years | 1) Generalised anxiety disorder  2) Panic disorder  3) Agoraphobia  4) Depression  5) Antisocial personality disorder  6) Substance-related disorder | 19-21 years | Moderate |
| Copeland et al. (2013) | New Zealand & USA | Survey-based longitudinal study | Community | 2,937 | - | 1) Anxiety disorder | Diagnostic assessment | 9-12 years | 1) Depression  2) Anxiety disorder  3) Conduct disorder  4) ADHD  5) Oppositional defiant disorder  6) Substance use disorder | 13-18 years | Strong |
| Copeland et al. (2014) | USA | Survey-based longitudinal study | Community | 1,420 | 51% female, 49% male | 1) Separation anxiety disorder  2) Social phobia  3) Generalised anxiety disorder  4) Overanxious disorder | Diagnostic assessment | 9-16 years | 1) Health functioning  2) Financial functioning  3) Interpersonal functioning | 19-26 years | Weak |
| Costello et al. (2003) | USA | Survey-based longitudinal study | Community | 1,420 | 45% female, 55% male | 1) Anxiety disorder | Diagnostic assessment | 9-13 years | 1) Depression  2) Anxiety disorder  3) Substance use disorder | 10-16 years | Strong |
| Dalsgaard et al. (2020) | Denmark | Register-based study | Community | 274,332 in female analysis, 268,168 in male analysis | Female, male | 1) Anxiety disorder | Medical records | Up to 16 years | 1) Final exam grade | Up to 17 years | Strong |
| Davies et al. (2016) | UK | Survey-based longitudinal study | Community | 2,835 | 55% female, 45% male | 1) Generalised anxiety disorder | Diagnostic assessment | 15 years | 1) Depression | 18 years | Moderate |
| de Hullu et al. (2017) | Netherlands | Longitudinal RCT | School | 70 | Female, male (proportions not specified) | 1) Elevated anxiety symptoms | Questionnaire with established cut-off score | 13 years (mean) | 1) Social phobia  2) Test anxiety  3) Self-esteem  4) Prosocial behaviours  5) Implicit associations  6) Fear of negative evaluation | 2 years after exposure | Weak |
| Dudani et al. (2010) | Canada | Survey-based longitudinal study | Community | 2,209,886 | 49% female, 51% male | 1) Elevated anxiety symptoms | Questionnaire with established cut-off score | 4-11 years | 1) Unintentional injury | 2 years after exposure | Strong |
| Dyer et al. (2019) | UK | Survey-based longitudinal study | Community | 3,462 | - | 1) Generalised anxiety disorder | Diagnostic assessment | 17 years | 1) Frequent drinking  2) Frequent bingeing  3) Hazardous drinking  4) Harmful drinking  5) Complete outcome data at follow-up | 20-21 years (median 20 years) | Moderate |
| Espejo et al. (2007) | Australia | Survey-based longitudinal study | Community | 816 | 49% female, 51% male | 1) Anxiety disorder | Diagnostic assessment | 0-14 years | 1) Depression severity index | 14 [0.4] years (mean [SD]) | Moderate |
| Essau et al. (2014) | USA | Survey-based longitudinal study | School | 816 | 59% female, 41% male | 1) Anxiety disorder | Diagnostic assessment | Up to 11 years | 1) High school completion  2) Unemployment  3) Annual household income  4) Physical health  5) Family support  6) Friend support  7) Total Social Adjustment Scale (SAS) score  8) Work SAS score  9) Leisure SAS score  10) Family SAS score  11) Marital role (SAS)  12) Parental role (SAS)  13) Family unit (SAS)  14) Suicide attempt  15) Life satisfaction  16) Coping skills  17) Chronic stress  18) Stressful life events | 29 [0.7] years (mean [SD]) | Strong |
| Fisher et al. (2016) | UK | Survey-based longitudinal study | Community | 1,831 | 61% female, 39% male | 1) Generalised anxiety disorder | Diagnostic assessment | 13 years | 1) Pain-related anxiety | 17 years | Weak |
| Fröjd et al. (2011) | Finland | Survey-based longitudinal study | School | 2,070 | 56% female, 44% male | 1) Elevated social phobia symptoms | Questionnaire with established cut-off score | 15-16 years | 1) Alcohol use  2) Drunkenness  3) Hash (cannabis) use | 17 [0.4] years (mean [SD]) | Strong |
| Gau et al. (2007) | Taiwan | Survey-based longitudinal study | School | 428 | 50% female, 50% male | 1) Anxiety disorder | Diagnostic assessment | 12 [0.3] years (mean [SD]) | 1) Substance use disorder | 2 years after exposure | Moderate |
| Ginsburg et al. (2018) | USA | Longitudinal RCT | Community | 224 | 55% female, 45% male | 1) Separation anxiety disorder  2) Social phobia  3) Generalised anxiety disorder | Diagnostic assessment | 7-17 years | 1) Anxiety remission (no anxiety diagnosis at any follow-up time) | 6 [1.65] years (mean [SD]) after exposure | Strong |
| Goldman-Mellor et al. (2014) | New Zealand | Survey-based longitudinal study | Community | 949 | 50% female, 50% male | 1) Anxiety disorder | Diagnostic assessment | 11-15 years | 1) Insomnia | 38 years | Strong |
| Goldstein et al. (2006) | USA | Survey-based longitudinal study | Community | 208 | Female, male (proportions not specified) | 1) Anxiety disorder | Diagnostic assessment | 6-17 years (mean [SD] 11 [3.2] years) | 1) Unmet mental health treatment needs | 24 [3.1] years (mean [SD]) | Strong |
| Goodwin et al. (2004) | New Zealand | Survey-based longitudinal study | Community | 1,025 | Female, male (proportions not specified) | 1) Panic attack | Diagnostic assessment | 15-18 years | 1) Psychoticism | 18 years | Strong |
| Goodwin et al. (2005) | USA | Survey-based longitudinal study | School | 836 | 57% female, 43% male | 1) Panic attack  2) Panic disorder | Diagnostic assessment | 14-18 years (mean [SD] 16 (1.2) years) | 1) Smoking | 24 years | Moderate |
| Griesler et al. (2008) | USA | Survey-based longitudinal study | School | 419 | 54% female, 46% male | 1) Anxiety disorder | Diagnostic assessment | 11-16 years | 1) Nicotine dependence disorder | 2 years after exposure | Strong |
| Griesler et al. (2011) | USA | Survey-based longitudinal study | School | 814 | 53% female, 47% male | 1) Anxiety disorder | Diagnostic assessment | At least 1 year pre outcome | 1) Nicotine dependence  3) Mood disorder  4) Disruptive disorder | 14 [1.6] years (mean [SD]) | Strong |
| Gundel et al. (2018) | Denmark | Register-based study | Community | 960,026 | 50% female, 50% male | 1) Anxiety disorder | Medical records | 5-17 years | 1) Depression | 9 years (mean) after exposure | Strong |
| Harpaz-Rotem et al. (2004) | USA | Register-based study | Community | 11,659 | 39% female, 61% male | 1) Anxiety disorder | Medical records | 11.51 (±4.0) years | 1) Treatment dropout  2) Number of mental health visits  3) Length of mental health visits | 6 months post exposure | Strong |
| Hayward et al. (2000) | USA | Survey-based longitudinal study | School | 2,365 | - | 1) Separation anxiety disorder | Diagnostic assessment | 15 (±0.9) years | 1) Panic attack  2) Major depression | Up to 4 years after exposure | Moderate |
| Hill et al. (2017) | USA | Survey-based longitudinal study | Community | 1,229 | Female, male (proportions not specified) | 1) Anxiety disorder | Diagnostic assessment | 9-16 years | 1) Cannabis involvement | 19-30 years | Moderate |
| Johnson et al. (2000) | USA | Survey-based longitudinal study | Community | 688 | 51% female, 49% male | 1) Anxiety disorder | Diagnostic assessment | 16 years (mean) | 1) Cigarette smoking | 22 years (mean) | Moderate |
| Kim-Cohen et al. (2003) | New Zealand | Survey-based longitudinal study | Community | 954 | 48% female, 52% male | 1) Anxiety disorder | Diagnostic assessment | 11-15 years | 1) Anxiety disorder  2) Depressive disorder  3) Substance use disorder  4) Manic episode  5) Schizophreniform disorder | 26 years | Moderate |
| King et al. (2004) | USA | Survey-based longitudinal study | Community | 699 | 100% female | 1) Separation anxiety disorder  2) Overanxious disorder | Diagnostic assessment | 10-12 years (mean 10 years) | 1) Alcohol use  2) Nicotine use  3) Cannabis use | 14 [0.5] years (mean [SD]) | Weak |
| Lloyd et al. (2020) | UK | Survey-based longitudinal study | Community | 2,406 | 100% female | 1) Anxiety disorder | Diagnostic assessment | 13-16 years | 1) Fasting for weight control | 13-16 years | Strong |
| Luby et al. (2009) | USA | Survey-based longitudinal study | Community | 256 | 48% female & 52% male | 1) Anxiety disorder | Diagnostic assessment | 3-5 years | 1) Major depressive disorder (MDD) | 2 years after exposure | Strong |
| Mars et al. (2014) | UK | Survey-based longitudinal study | Community | 4,799 | 59% female, 41% male | 1) Anxiety disorder | Diagnostic assessment | 15 years | 1) Self-harm without suicidal intent  2) Self-harm with suicidal intent | 16-17 years | Weak |
| Mars et al. (2019) | UK | Survey-based longitudinal study | Community | 4,772 | 58% female, 42% male | 1) Anxiety disorder | Diagnostic assessment | 15 years | 1) Suicide ideation and attempts | 16 years | Weak |
| Mathew et al. (2011) | USA | Survey-based longitudinal study | School | 1,507 | 59% female, 41% male | 1) Anxiety disorder | Diagnostic assessment | 14-18 years (mean [SD] 16 [1.2] years) | 1) Major depressive disorder | Up to 30 years | Strong |
| Olino et al. (2008) | USA | Survey-based longitudinal study | School | 891 | 57% female, 43% male | 1) Anxiety disorder | Diagnostic assessment | Up to 18 years | 1) Depressive disorder  2) Anxiety disorder | 14-30 years | Moderate |
| Ormel et al. (2015) | Netherlands | Survey-based longitudinal study | Community | 1,584 | 54% female, 46% male | 1) Elevated anxiety symptoms | Questionnaire with established cut-off score | 11 [0.6] years (mean [SD]) | 1) Anxiety disorder  2) Behaviour disorder  3) Mood disorder  4) Substance dependence | 18-20 years (mean [SD] 19 [0.6] years) | Strong |
| Perez et al. (2007) | Spain | Survey-based longitudinal study | Community | 126 | 46% female, 54% male | 1) Anxiety disorder | Diagnostic assessment | 11-15 years | 1) Study drop-out | 1 year after exposure | Weak |
| Ranoyen et al. (2018) | Norway | Survey-based longitudinal study | Health care | 549 | 56% female, 44% male | 1) Anxiety disorder  2) Generalised anxiety disorder  3) Social anxiety disorder  4) Agora- and specific phobia | Medical records | 13-18 years | 1) Anxiety disorder  2) Depression  3) Generalised anxiety disorder  4) Social phobia  5) Agora- and specific phobia | 16-21 years (mean 18 years) | Moderate |
| Ranta et al. (2016) | Finland | Survey-based longitudinal study | School | 2,070 | 56% female, 44% male | 1) Social phobia | Questionnaire with established cut-off score | 15 [0.4] years (mean [SD]) | 1) Grade point average  2) School/work dropout  3) School absence  4) Academic progress  5) Close friends  6) Romantic partners  7) Friendship support  8) Partner support | 17 [0.4] years (mean [SD]) | Strong |
| Ranta et al. (2017) | Finland | Survey-based longitudinal study | School | 2,070 | 56% female, 44% male | 1) Social phobia | Questionnaire with established cut-off score | 15 [0.4] years (mean [SD]) | 1) Bulimia nervosa  2) Anorexia nervosa  3) Social phobia  4) Not sought treatment for eating disorder | 17 [0.4] years (mean [SD]) | Strong |
| Reef et al. (2010) | Netherlands | Survey-based longitudinal study | Community | 1,339 | Female, male (proportions not specified) | 1) Elevated anxiety symptoms | Questionnaire with established cut-off score | 4-16 years | 1) Anxiety disorder  2) Mood disorder  3) Substance use disorder  4) Disruptive disorder  5) Any psychiatric disorder | 28-40 years | Strong |
| Rofey et al. (2009) | - | Survey-based longitudinal study | - | 285 | 49% female, 51% male | 1) Generalised anxiety disorder | Diagnostic assessment | 8-18 years (mean [SD] 11 [2.3] years) | 1) Body mass index | 11-21 years | Moderate |
| Shanahan et al. (2014) | USA | Survey-based longitudinal study | Community | 1,420 | Female, male (proportions not specified) | 1) Generalised anxiety disorder  2) Separation anxiety disorder | Diagnostic assessment | 9-13 years | 1) Sleep problems | 9-16 years | Strong |
| Shevlin et al. (2017) | UK | Survey-based longitudinal study | Community | 4,815 | - | 1) Specific phobia  2) Social phobia  3) Generalised anxiety disorder | Diagnostic assessment | 7 years | 1) Specific phobia  2) Social phobia  3) Generalised anxiety disorder  4) Depression | 14 years | Strong |
| Sihvola et al. (2009) | Finland | Survey-based longitudinal study | Community | 1,318 | 49% female, 51% male | 1) Generalised anxiety disorder | Diagnostic assessment | 14 years | 1) Eating disorder | 17 years | Strong |
| Sung et al. (2004) | USA | Survey-based longitudinal study | Community | 1,420 | - | 1) Anxiety disorder | Diagnostic assessment | 0-16 years | 1) Substance use disorder | 13-16 years | Strong |
| Thirlwall et al. (2013) | UK | Longitudinal RCT | Community | 69 | 49% female, 51% male | 1) Anxiety disorder | Medical records | 7-12 years | 1) Spence Children’s Anxiety Scale  2) Child Anxiety Impact Scale  3) Short Moods and Feelings Questionnaire  4) Strengths and Difficulties Questionnaire | 12 weeks after exposure | Strong |
| Vaananen et al. (2011) | Finland | Survey-based longitudinal study | School | 1,017 in female analysis, 824 in male analysis | Female, male | 1) Social phobia | Questionnaire with established cut-off score | 15 [0.4] years (mean [SD]) | 1) Depression | 17 [0.4] years (mean [SD]) | Strong |
| Vaananen et al. (2014) | Finland | Survey-based longitudinal study | School | 1,021 in female analysis, 831 in male analysis | Female, male | 1) Social phobia | Questionnaire with established cut-off score | 15 [0.4] years (mean [SD]) | 1) Depression  2) Self-esteem | 17 [0.4] years (mean [SD]) | Strong |
| Virtanen et al. (2021) | Sweden | Survey-based longitudinal study | Community | 1,768,516 | 49% female, 51% male | 1) Elevated anxiety symptoms  2) Anxiety disorder | Questionnaire with established cut-off score | 0-12 years | 1) Substance misuse | 17.2-18.4 (median) years | Strong |
| Warner et al. (2005) | USA | Register-based study | Community | 2,749 | 37% female, 63% male | 1) Anxiety disorder | Medical records | 0-17 years | 1) Psychotropic prescription | Up to 1 year post exposure | Strong |
| Wittchen et al. (2007) | Germany | Survey-based longitudinal study | Community | 1,310 | - | 1) Anxiety disorder  2) Panic attack  3) Social phobia  4) Specific phobia  5) Generalised anxiety disorder  6) Agoraphobia  7) Separation anxiety disorder | Diagnostic assessment | 14-17 years | 1) Cannabis use  2) Cannabis use disorder | 21-27 years | Strong |
| Wolitzky-Taylor et al. (2012) | USA | Survey-based longitudinal study | School | 627 | 69% female, 31% male | 1) Anxiety disorder  2) Social phobia | Diagnostic assessment | 16 [0.4] years (mean [SD]) | 1) Alcohol use disorder  2) Non-alcohol use disorder  3) Substance use disorder | 1-4 years after exposure | Moderate |
| Woodward and Fergusson (2001) | New Zealand | Survey-based longitudinal study | Community | 964 | 50% female, 50% male | 1) Anxiety disorder | Diagnostic assessment | 14-16 years | 1) Anxiety disorder  2) Major depression disorder  3) Nicotine dependence  4) Alcohol abuse/ dependence  5) Illicit drug dependence  6) Suicide attempt  7) University enrolment  8) Tertiary education/ training enrolment  9) Became a parent  10) Unemployed | 16-21 years | Strong |
| Wuthrich et al. (2012) | Australia | Longitudinal RCT | Community | 19 | 58% female, 42% male | 1) Anxiety disorder | Diagnostic assessment | 14-17 years (mean [SD] 15 [1.1] years) | 1) Number of anxiety disorders  2) Severity of main disorder  3) Mean severity of all disorder  4) Spence Child Anxiety Scale  5) Strengths and Difficulties Questionnaire Emotional Symptoms subscale  6) Adolescent Life Interference Scale  7) Children’s Automatic Thoughts Questionnaire | 12 weeks after exposure | Strong |
| Yoshimasu et al. (2016) | USA | Survey-based longitudinal study | Community | 1,055 | 48% female, 52% male | 1) Anxiety disorder | Medical records | 0-18 years | 1) Substance use disorder | 0-19 years | Moderate |
| Zaider et al. (2002) | USA | Survey-based longitudinal study | School & health care | 201 | 78% female, 22% male | 1) Generalised anxiety disorder  2) Panic disorder | Questionnaire with established cut-off score | 15-18 years (mean [SD] 16 [1.1] years) | 1) Bulimia nervosa or binge eating disorder  2) Bulimia nervosa or binge eating symptoms | 10 months after exposure | Moderate |

^1^ Largest sample size from all included analyses in study; ^2^ Most inclusive analysis; ^3^ Range across all included analyses in study; ^4^ Analysis with most controls; - no information; EPHPP = Effective Public Health Practice Project; SES = socioeconomic status; ADHD = attention deficit hyperactivity disorder; RCT = randomised controlled trial.

Figure S1: Proportion of associations and strength of evidence between anxiety exposure (by age category) and each outcome domain


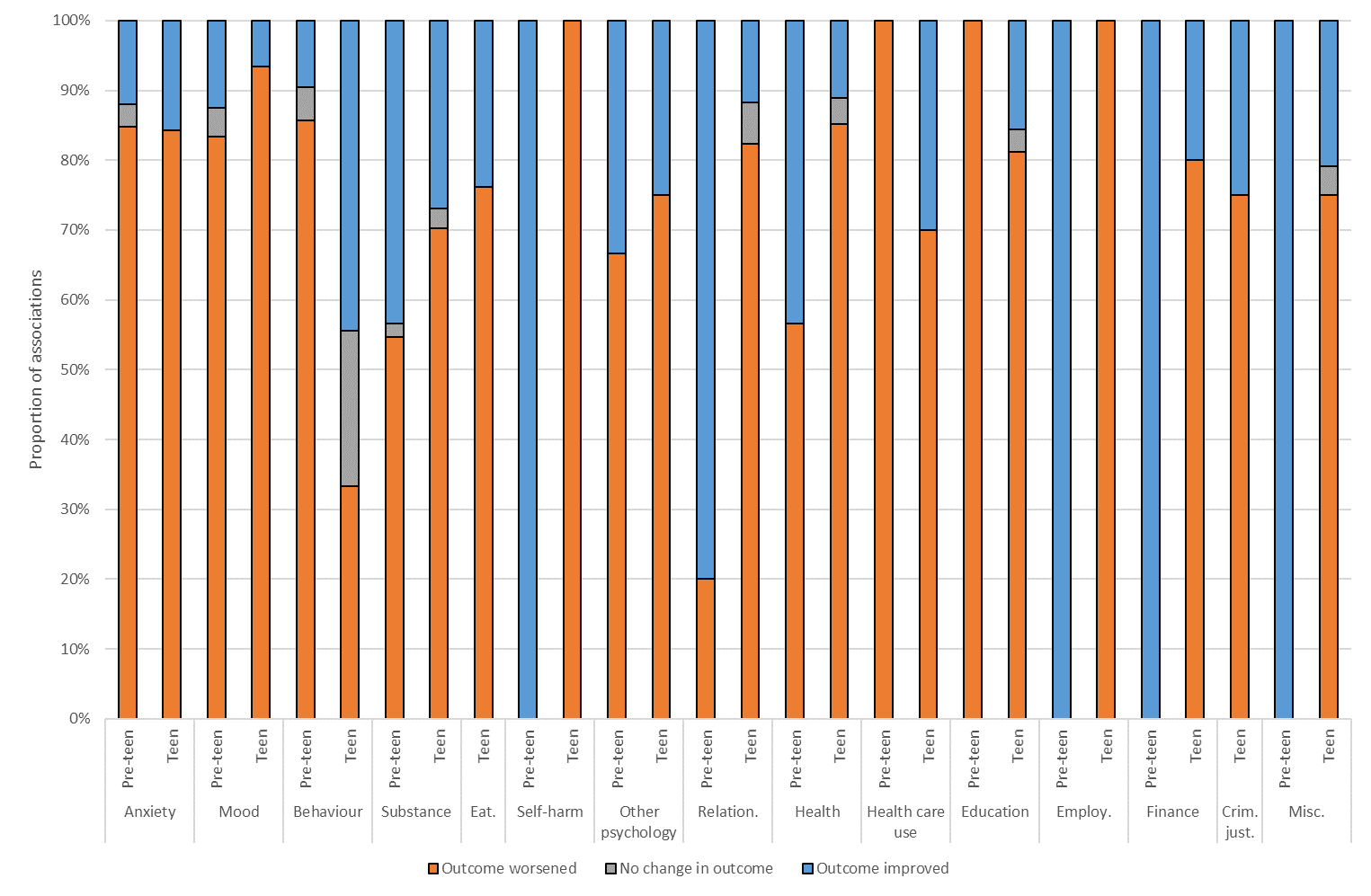


All outcome age categories (pre-teenage, teenage, adult) grouped together.

Figure S2: Meta-analyses of the association between childhood anxiety and subsequent mood outcomes

| **A: Child anxiety disorder (exposure)** $\boldsymbol{\to}$ **Child depressive disorder (outcome)**  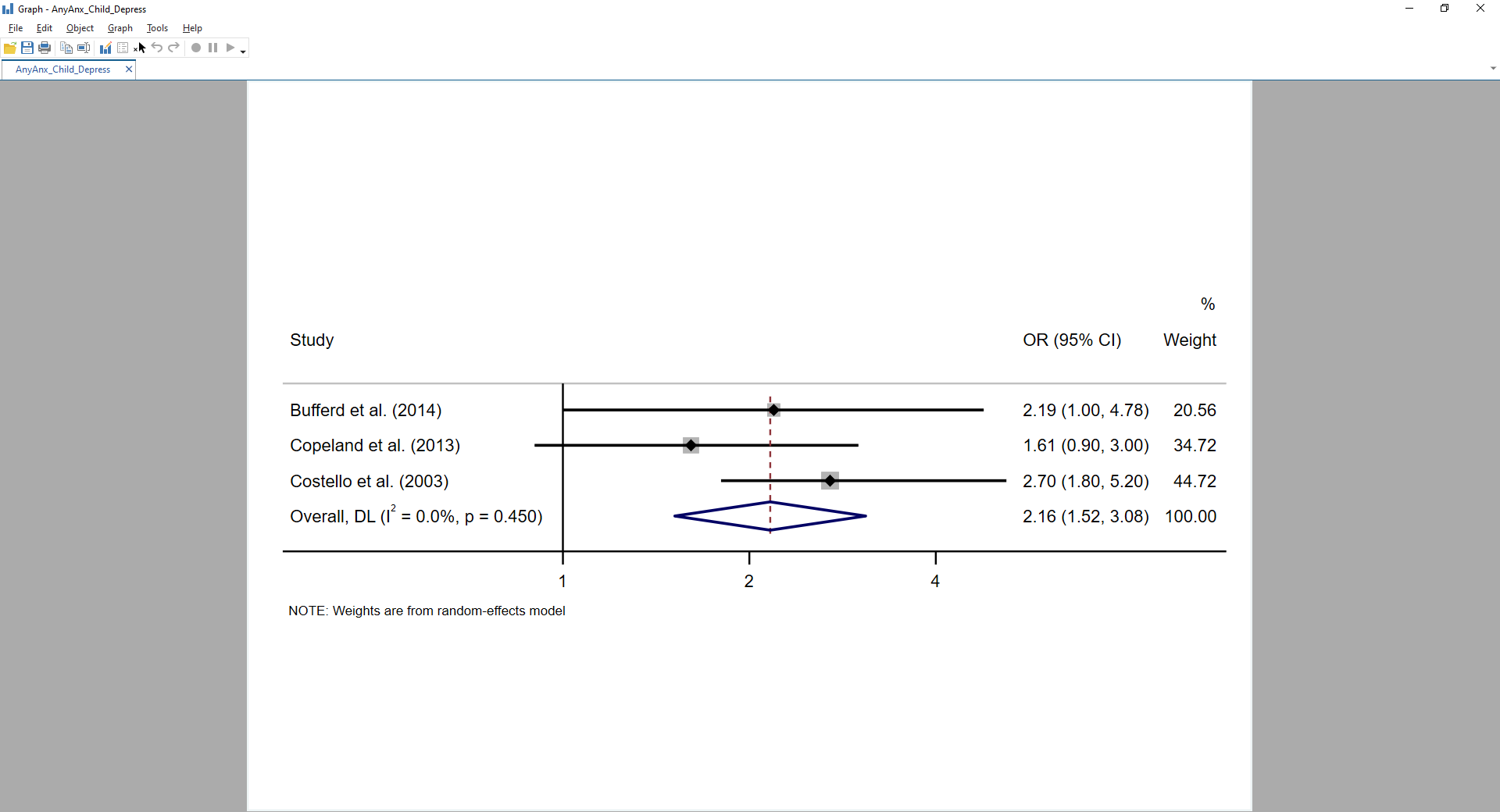 | **B: Child anxiety disorder (exposure)** $\boldsymbol{\to}$ **Adult depressive disorder (outcome)**  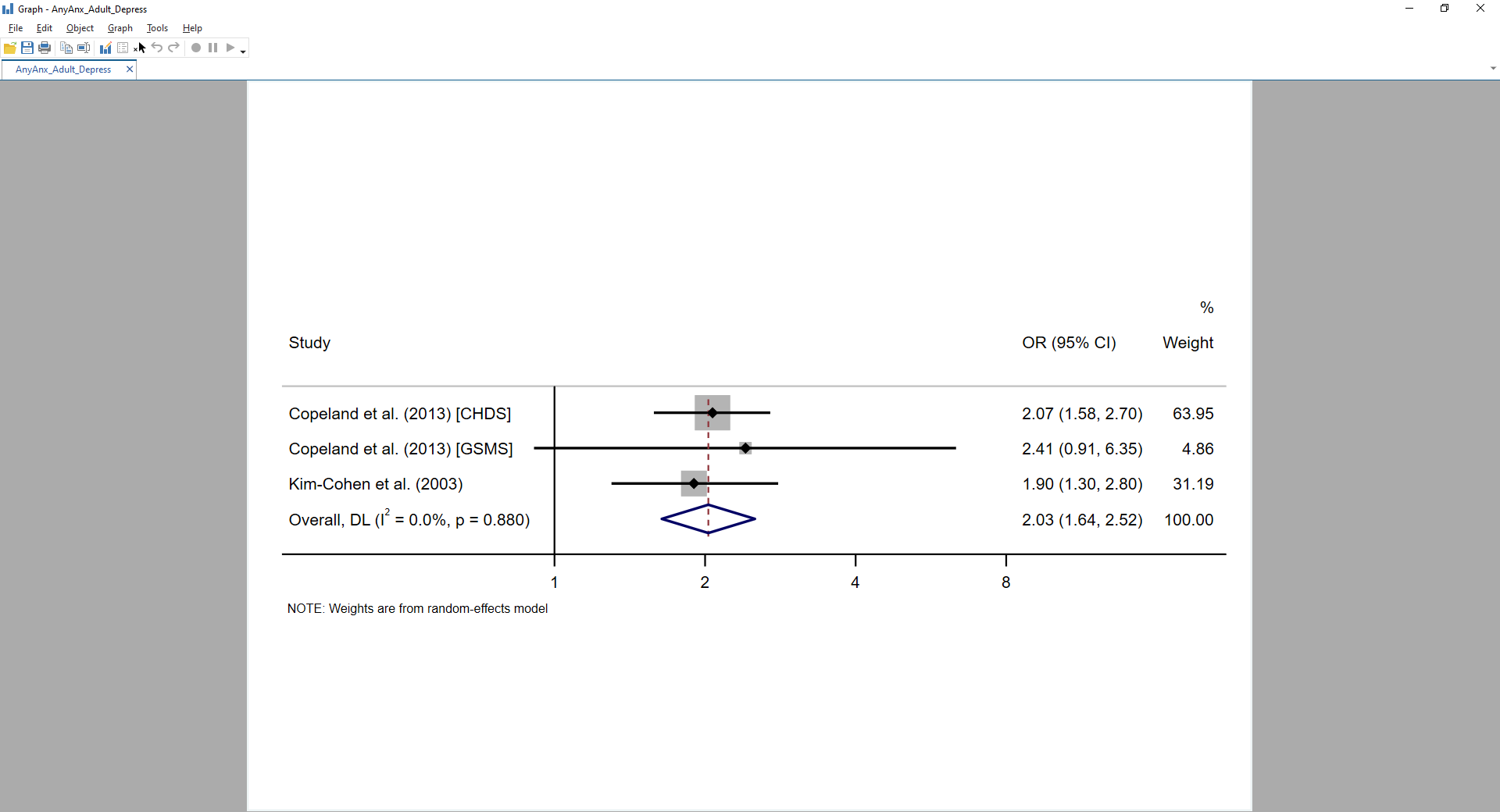  CHDS=Christchurch Health and Development Study; GSMS=Great Smoky Mountain Study. |
| --- | --- |
| **C: Child separation anxiety disorder (exposure)** $\boldsymbol{\to}$ **Child depressive disorder (outcome)**  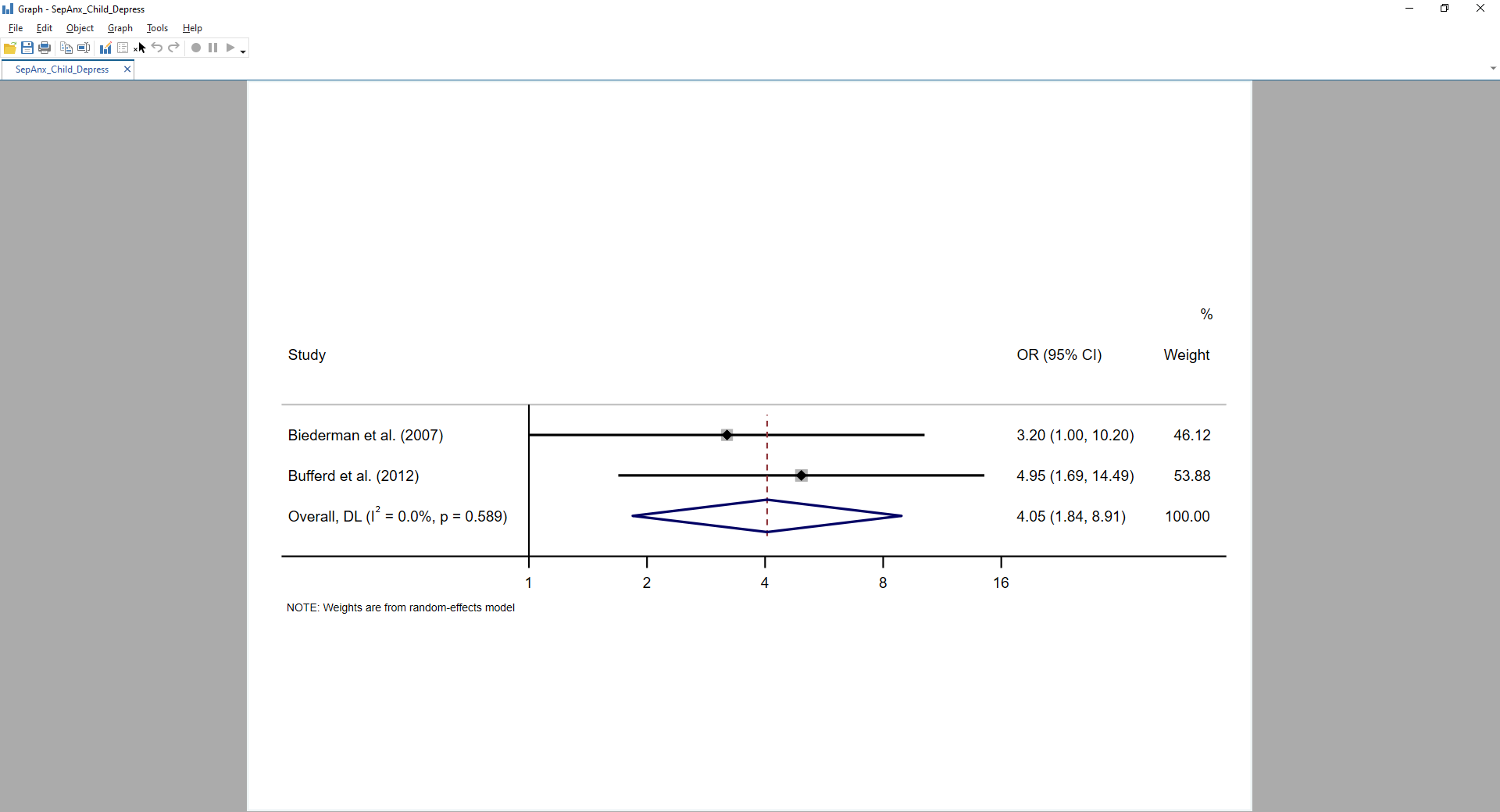 |  |

Figure S3: Meta-analyses of the association between childhood anxiety and subsequent behaviour outcomes

| **A: Child anxiety disorder (exposure)** $\boldsymbol{\to}$ **Child ADHD (outcome)**  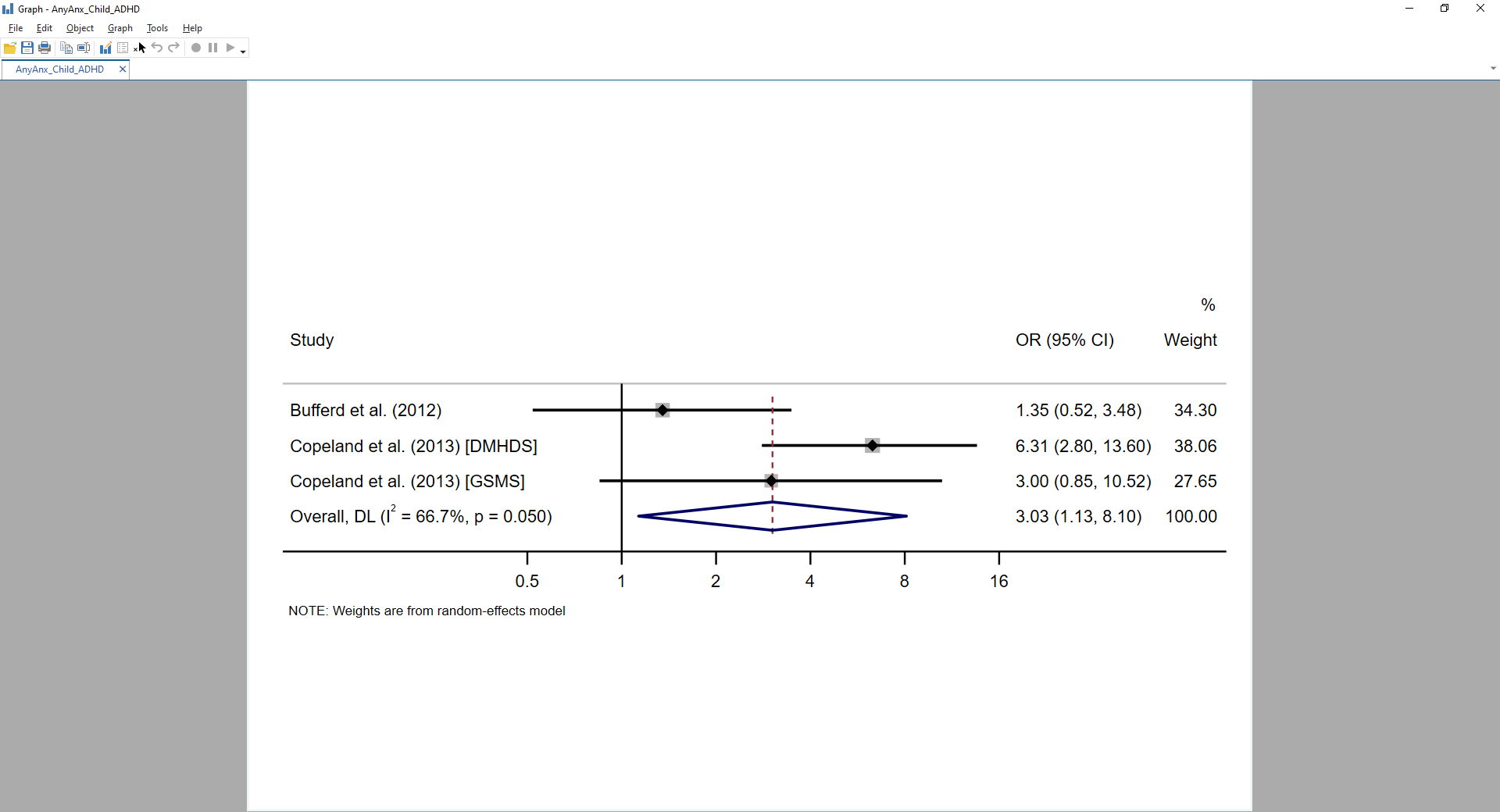  DMHDS=Dunedin Multidisciplinary Health and Development Study; GSMS=Great Smoky Mountain Study. | **B: Child anxiety disorder (exposure)** $\boldsymbol{\to}$ **Child ODD (outcome)**  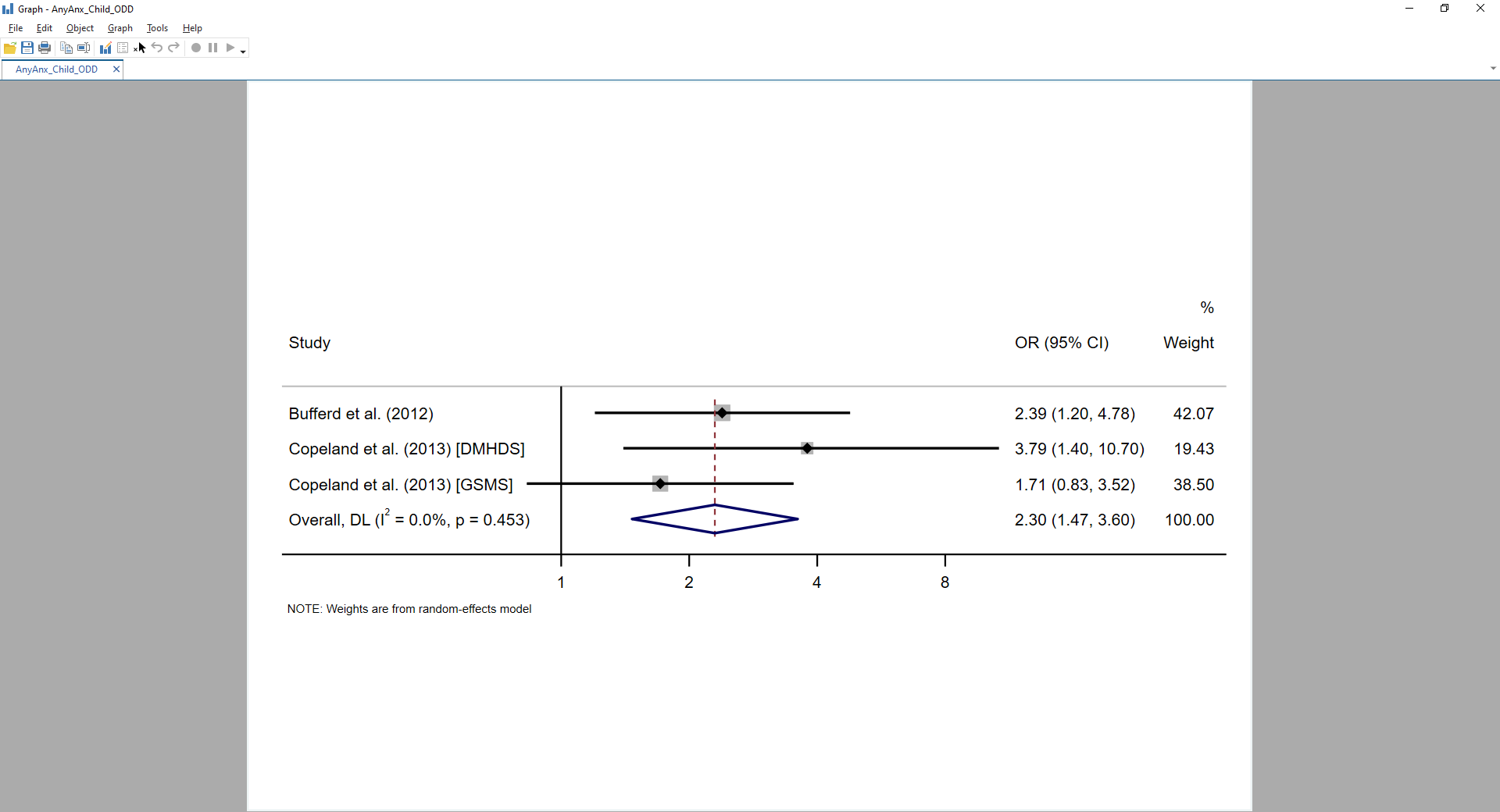  DMHDS=Dunedin Multidisciplinary Health and Development Study; GSMS=Great Smoky Mountain Study. |
| --- | --- |

Figure S4: Meta-analyses of the association between childhood anxiety and subsequent substance use outcomes

| **A: Child anxiety disorder (exposure)** $\boldsymbol{\to}$ **Child substance use disorder (outcome)**  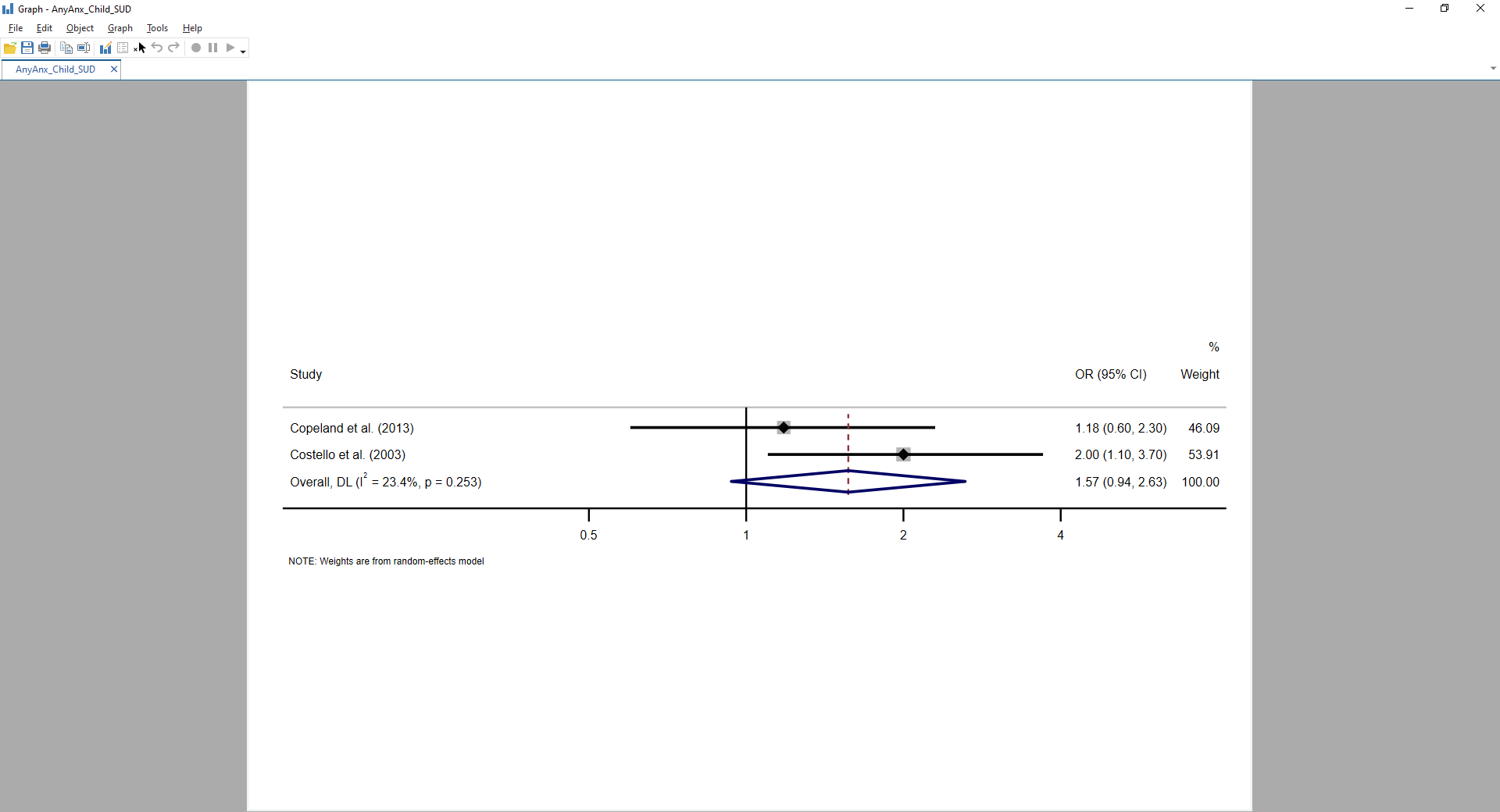 | **B: Child anxiety disorder (exposure)** $\boldsymbol{\to}$ **Adult substance use disorder (outcome)**  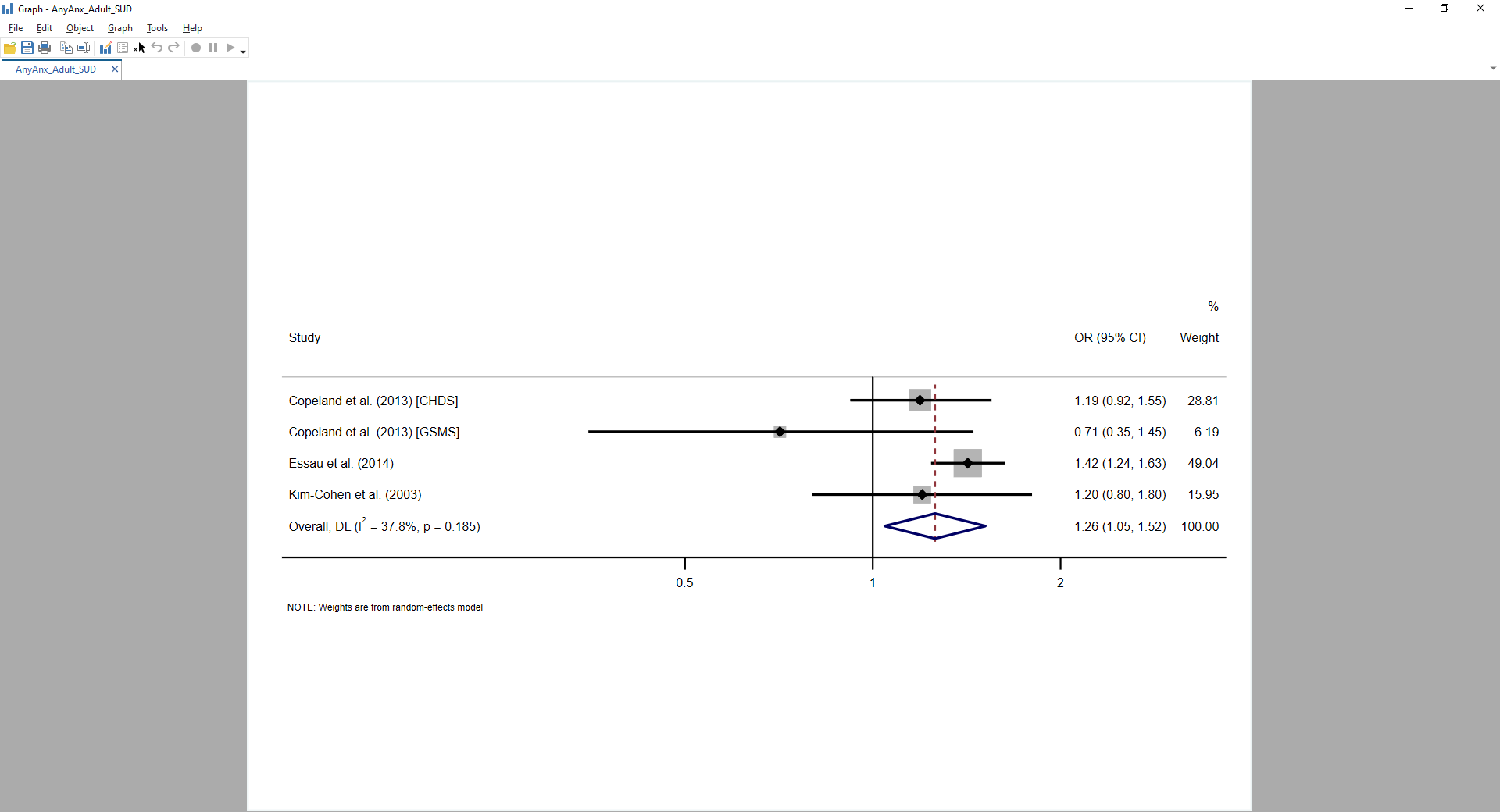  CHDS=Christchurch Health and Development Study; GSMS=Great Smoky Mountain Study. |
| --- | --- |
|  |  |

**References**

Aarons, G. A., Monn, A. R., Leslie, L. K., Garland, A. F., Lugo, L., Hough, R. L., et al. (2008). Association between mental and physical health problems in high-risk adolescents: a longitudinal study. [Research Support, N.I.H., Extramural]. *Journal of Adolescent Health, 43*(3), 260-267.

Ali, M. M., Teich, J., Lynch, S., & Mutter, R. (2018). Utilization of mental health services by preschool-aged children with private insurance coverage. *Administration and Policy in Mental Health and Mental Health Services Research, 45*(5), 731-740.

Aparicio, E., Canals, J., Voltas, N., Hernandez-Martinez, C., & Arija, V. (2013). Emotional psychopathology and increased adiposity: Follow-up study in adolescents. *Journal of Adolescence, 36*(2), 319-330.

Aschenbrand, S. G., Kendall, P. C., Webb, A., Safford, S. M., & Flannery-Schroeder, E. (2003). Is childhood separation anxiety disorder a predictor of adult panic disorder and agoraphobia? A seven-year longitudinal study. *Journal of the American Academy of Child & Adolescent Psychiatry, 42*(12), 1478-1485.

Belden, A. C., Gaffrey, M. S., & Luby, J. L. (2012). Relational aggression in children with preschool-onset psychiatric disorders. [Research Support, N.I.H., Extramural]. *Journal of the American Academy of Child & Adolescent Psychiatry, 51*(9), 889-901.

Biederman, J., Petty, C. R., Hirshfeld-Becker, D. R., Henin, A., Faraone, S. V., Fraire, M., et al. (2007). Developmental trajectories of anxiety disorders in offspring at high risk for panic disorder and major depression. [Comparative Study Research Support, N.I.H., Extramural]. *Psychiatry Research, 153*(3), 245-252.

Bittner, A., Egger, H. L., Erkanli, A., Jane Costello, E., Foley, D. L., & Angold, A. (2007). What do childhood anxiety disorders predict? *Journal of Child Psychology and psychiatry, 48*(12), 1174-1183.

Bodden, D. H., Dirksen, C. D., & Bogels, S. M. (2008). Societal burden of clinically anxious youth referred for treatment: a cost-of-illness study. [Comparative Study Multicenter Study Randomized Controlled Trial Research Support, Non-U.S. Gov't]. *Journal of Abnormal Child Psychology, 36*(4), 487-497.

Bodden, D. H. M., Dirksen, C. D., Bogels, S. M., Nauta, M. H., De Haan, E., Ringrose, J., et al. (2008). Costs and cost-effectiveness of family CBT versus individual CBT in clinically anxious children. *Clinical Child Psychology and Psychiatry\, 13*(4), 543-564\.

Bohnert, A. M., & Garber, J. (2007). Prospective relations between organized activity participation and psychopathology during adolescence. [Research Support, N.I.H., Extramural Research Support, Non-U.S. Gov't]. *Journal of Abnormal Child Psychology, 35*(6), 1021-1033.

Buckner, J. D., Schmidt, N. B., Lang, A. R., Small, J. W., Schlauch, R. C., & Lewinsohn, P. M. (2008). Specificity of social anxiety disorder as a risk factor for alcohol and cannabis dependence. [Research Support, N.I.H., Extramural Research Support, Non-U.S. Gov't]. *Journal of Psychiatric Research, 42*(3), 230-239.

Bufferd, S. J., Dougherty, L. R., Carlson, G. A., Rose, S., & Klein, D. N. (2012). Psychiatric disorders in preschoolers: continuity from ages 3 to 6. *American Journal of Psychiatry, 169*(11), 1157-1164.

Bufferd, S. J., Dougherty, L. R., Olino, T. M., Dyson, M. W., Laptook, R. S., Carlson, G. A., et al. (2014). Predictors of the onset of depression in young children: a multi-method, multi-informant longitudinal study from ages 3 to 6. [Research Support, N.I.H., Extramural Research Support, Non-U.S. Gov't]. *Journal of Child Psychology & Psychiatry & Allied Disciplines, 55*(11), 1279-1287.

Burke, J. D., Loeber, R., Lahey, B. B., & Rathouz, P. J. (2005). Developmental transitions among affective and behavioral disorders in adolescent boys. *Journal of Child Psychology and Psychiatry, 46*(11), 1200-1210.

Chatterton, M. L., Rapee, R. M., Catchpool, M., Lyneham, H. J., Wuthrich, V., Hudson, J. L., et al. (2019). Economic evaluation of stepped care for the management of childhood anxiety disorders: Results from a randomised trial. *Australian & New Zealand Journal of Psychiatry, 53*(7), 673-682.

Chen, H., Cohen, P., Crawford, T., Kasen, S., Guan, B., & Gorden, K. (2009). Impact of early adolescent psychiatric and personality disorder on long-term physical health: A 20-year longitudinal follow-up study. *Psychological Medicine, 39*(5), 865-874.

Chen, H., Cohen, P., Kasen, S., Johnson, J. G., Berenson, K., & Gordon, K. (2006). Impact of adolescent mental disorders and physical illnesses on quality of life 17 years later. [Research Support, N.I.H., Extramural]. *Archives of Pediatrics & Adolescent Medicine, 160*(1), 93-99.

Copeland, W. E., Adair, C. E., Smetanin, P., Stiff, D., Briante, C., Colman, I., et al. (2013). Diagnostic transitions from childhood to adolescence to early adulthood. [Research Support, N.I.H., Extramural Research Support, Non-U.S. Gov't]. *Journal of Child Psychology & Psychiatry & Allied Disciplines, 54*(7), 791-799.

Copeland, W. E., Angold, A., Shanahan, L., & Costello, E. J. (2014). Longitudinal patterns of anxiety from childhood to adulthood: the Great Smoky Mountains Study. [Research Support, N.I.H., Extramural Research Support, Non-U.S. Gov't Review]. *Journal of the American Academy of Child & Adolescent Psychiatry, 53*(1), 21-33.

Copeland, W. E., Miller-Johnson, S., Keeler, G., Angold, A., & Costello, E. J. (2007). Childhood psychiatric disorders and young adult crime: a prospective, population-based study. [Research Support, N.I.H., Extramural Research Support, Non-U.S. Gov't]. *American Journal of Psychiatry, 164*(11), 1668-1675.

Copeland, W. E., Shanahan, L., Costello, E. J., & Angold, A. (2009). Childhood and adolescent psychiatric disorders as predictors of young adult disorders. [Research Support, N.I.H., Extramural Research Support, Non-U.S. Gov't]. *Archives of General Psychiatry, 66*(7), 764-772.

Costello, E. J., Copeland, W., Cowell, A., & Keeler, G. (2007). Service costs of caring for adolescents with mental illness in a rural community, 1993-2000. [Comparative Study Research Support, N.I.H., Extramural Research Support, Non-U.S. Gov't]. *American Journal of Psychiatry, 164*(1), 36-42.

Costello, E. J., Mustillo, S., Erkanli, A., Keeler, G., & Angold, A. (2003). Prevalence and development of psychiatric disorders in childhood and adolescence. [Research Support, Non-U.S. Gov't Research Support, U.S. Gov't, P.H.S.]. *Archives of General Psychiatry, 60*(8), 837-844.

Creswell, C., Violato, M., Cruddace, S., Gerry, S., Murray, L., Shafran, R., et al. (2020). A randomised controlled trial of treatments of childhood anxiety disorder in the context of maternal anxiety disorder: clinical and cost-effectiveness outcomes. *Journal of Child Psychology & Psychiatry & Allied Disciplines, 61*(1), 62-76.

Creswell, C., Violato, M., Fairbanks, H., White, E., Parkinson, M., Abitabile, G., et al. (2017). Clinical outcomes and cost-effectiveness of brief guided parent-delivered cognitive behavioural therapy and solution-focused brief therapy for treatment of childhood anxiety disorders: a randomised controlled trial. [Comparative Study Randomized Controlled Trial Research Support, Non-U.S. Gov't]. *The Lancet. Psychiatry, 4*(7), 529-539.

Dalsgaard, S., McGrath, J., Ostergaard, S. D., Wray, N. R., Pedersen, C. B., Mortensen, P. B., et al. (2020). Association of Mental Disorder in Childhood and Adolescence With Subsequent Educational Achievement. *JAMA Psychiatry, 25*, 25.

Davies, S. J., Pearson, R. M., Stapinski, L., Bould, H., Christmas, D. M., Button, K. S., et al. (2016). Symptoms of generalized anxiety disorder but not panic disorder at age 15 years increase the risk of depression at 18 years in the Avon Longitudinal Study of Parents and Children (ALSPAC) cohort study. [Research Support, Non-U.S. Gov't]. *Psychological Medicine, 46*(1), 73-85.

de Hullu, E., Sportel, B. E., Nauta, M. H., & de Jong, P. J. (2017). Cognitive bias modification and CBT as early interventions for adolescent social and test anxiety: Two-year follow-up of a randomized controlled trial. [Multicenter Study Randomized Controlled Trial]. *Journal of Behavior Therapy & Experimental Psychiatry, 55*, 81-89.

Dudani, A., Macpherson, A., & Tamim, H. (2010). Childhood behavior problems and unintentional injury: a longitudinal, population-based study. [Research Support, Non-U.S. Gov't]. *Journal of Developmental & Behavioral Pediatrics, 31*(4), 276-285.

Dyer, M. L., Heron, J., Hickman, M., & Munafo, M. R. (2019). Alcohol use in late adolescence and early adulthood: The role of generalized anxiety disorder and drinking to cope motives. *Drug and Alcohol Dependence Vol 204 2019, ArtID 107480, 204*.

Espejo, E. P., Hammen, C. L., Connolly, N. P., Brennan, P. A., Najman, J. M., & Bor, W. (2007). Stress sensitization and adolescent depressive severity as a function of childhood adversity: a link to anxiety disorders. *Journal of Abnormal Child Psychology, 35*(2), 287-299.

Essau, C. A., Lewinsohn, P. M., Olaya, B., & Seeley, J. R. (2014). Anxiety disorders in adolescents and psychosocial outcomes at age 30. [Research Support, N.I.H., Extramural Research Support, Non-U.S. Gov't]. *Journal of Affective Disorders, 163*, 125-132.

Fisher, E., Caes, L., Clinch, J., Tobias, J. H., & Eccleston, C. (2016). Anxiety at 13 and its effect on pain, pain-related anxiety, and pain-related disability at 17: An ALSPAC cohort longitudinal analysis. [Research Support, Non-U.S. Gov't]. *Psychology Health & Medicine, 21*(1), 1-9.

Fröjd, S., Ranta, K., Kaltiala-Heino, R., & Marttunen, M. (2011). Associations of social phobia and general anxiety with alcohol and drug use in a community sample of adolescents. *Alcohol and alcoholism, 46*(2), 192-199.

Gau, S. S., Chong, M. Y., Yang, P., Yen, C. F., Liang, K. Y., & Cheng, A. T. (2007). Psychiatric and psychosocial predictors of substance use disorders among adolescents: longitudinal study. [Research Support, Non-U.S. Gov't]. *British Journal of Psychiatry, 190*, 42-48.

Ginsburg, G. S., Becker-Haimes, E. M., Keeton, C., Kendall, P. C., Iyengar, S., Sakolsky, D., et al. (2018). Results From the Child/Adolescent Anxiety Multimodal Extended Long-Term Study (CAMELS): Primary Anxiety Outcomes. [Research Support, N.I.H., Extramural]. *Journal of the American Academy of Child & Adolescent Psychiatry, 57*(7), 471-480.

Goldman-Mellor, S., Gregory, A. M., Caspi, A., Harrington, H., Parsons, M., Poulton, R., et al. (2014). Mental health antecedents of early midlife insomnia: evidence from a four-decade longitudinal study. *Sleep, 37*(11), 1767-1775.

Goldstein, R. B., Olfson, M., Martens, E. G., & Wolk, S. I. (2006). Subjective unmet need for mental health services in depressed children grown up. [Research Support, N.I.H., Extramural Research Support, Non-U.S. Gov't]. *Administration & Policy in Mental Health, 33*(6), 666-673.

Goodwin, R. D., Fergusson, D. M., & Horwood, L. J. (2004). Panic attacks and psychoticism. [Research Support, Non-U.S. Gov't]. *American Journal of Psychiatry, 161*(1), 88-92.

Goodwin, R. D., Lewinsohn, P. M., & Seeley, J. R. (2005). Cigarette smoking and panic attacks among young adults in the community: the role of parental smoking and anxiety disorders. *Biological psychiatry, 58*(9), 686-693.

Griesler, P. C., Hu, M. C., Schaffran, C., & Kandel, D. B. (2008). Comorbidity of psychiatric disorders and nicotine dependence among adolescents: findings from a prospective, longitudinal study. [Research Support, N.I.H., Extramural Research Support, Non-U.S. Gov't]. *Journal of the American Academy of Child & Adolescent Psychiatry, 47*(11), 1340-1350.

Griesler, P. C., Hu, M. C., Schaffran, C., & Kandel, D. B. (2011). Comorbid psychiatric disorders and nicotine dependence in adolescence. [Research Support, N.I.H., Extramural]. *Addiction, 106*(5), 1010-1020.

Gundel, L. K., Pedersen, C. B., Munk-Olsen, T., & Dalsgaard, S. (2018). Longitudinal association between mental disorders in childhood and subsequent depression - A nationwide prospective cohort study. [Research Support, Non-U.S. Gov't]. *Journal of Affective Disorders, 227*, 56-64.

Harpaz-Rotem, I., Leslie, D., & Rosenheck, R. A. (2004). Treatment retention among children entering a new episode of mental health care. *Psychiatric Services, 55*(9), 1022-1028.

Hayward, C., Killen, J. D., Kraemer, H. C., & Taylor, C. B. (2000). Predictors of panic attacks in adolescents. *Journal of the American Academy of Child & Adolescent Psychiatry, 39*(2), 207-214.

Hill, S., Shanahan, L., Costello, E. J., & Copeland, W. (2017). Predicting Persistent, Limited, and Delayed Problematic Cannabis Use in Early Adulthood: Findings From a Longitudinal Study. *Journal of the American Academy of Child & Adolescent Psychiatry, 56*(11), 966-974.e964.

Johnson, J. G., Cohen, P., Pine, D. S., Klein, D. F., Kasen, S., & Brook, J. S. (2000). Association between cigarette smoking and anxiety disorders during adolescence and early adulthood. [Research Support, U.S. Gov't, P.H.S.]. *JAMA, 284*(18), 2348-2351.

Kim-Cohen, J., Caspi, A., Moffitt, T. E., Harrington, H., Milne, B. J., & Poulton, R. (2003). Prior juvenile diagnoses in adults with mental disorder: developmental follow-back of a prospective-longitudinal cohort. [Comparative Study Research Support, Non-U.S. Gov't Research Support, U.S. Gov't, P.H.S.]. *Archives of General Psychiatry, 60*(7), 709-717.

King, S. M., Iacono, W. G., & McGue, M. (2004). Childhood externalizing and internalizing psychopathology in the prediction of early substance use. [Research Support, U.S. Gov't, P.H.S. Twin Study]. *Addiction, 99*(12), 1548-1559.

Libutzki, B., Ludwig, S., May, M., Jacobsen, R. H., Reif, A., & Hartman, C. A. (2019). Direct medical costs of ADHD and its comorbid conditions on basis of a claims data analysis. [Research Support, Non-U.S. Gov't]. *European Psychiatry: the Journal of the Association of European Psychiatrists, 58*, 38-44.

Lim, D., Lee, W. K., & Park, H. (2016). Disability-adjusted Life Years (DALYs) for Mental and Substance Use Disorders in the Korean Burden of Disease Study 2012. *Journal of Korean Medical Science, 31 Suppl 2*, S191-S199.

Lloyd, E. C., Haase, A. M., Zerwas, S., & Micali, N. (2020). Anxiety disorders predict fasting to control weight: A longitudinal large cohort study of adolescents. *European Eating Disorders Review, 28*(3), 269-281.

Luby, J. L., Si, X., Belden, A. C., Tandon, M., & Spitznagel, E. (2009). Preschool depression: homotypic continuity and course over 24 months. [Comparative Study Research Support, N.I.H., Extramural]. *Archives of General Psychiatry, 66*(8), 897-905.

Mars, B., Heron, J., Crane, C., Hawton, K., Kidger, J., Lewis, G., et al. (2014). Differences in risk factors for self-harm with and without suicidal intent: findings from the ALSPAC cohort. [Research Support, Non-U.S. Gov't]. *Journal of Affective Disorders, 168*, 407-414.

Mars, B., Heron, J., Klonsky, E. D., Moran, P., O'Connor, R. C., Tilling, K., et al. (2019). What distinguishes adolescents with suicidal thoughts from those who have attempted suicide? A population-based birth cohort study. [Research Support, Non-U.S. Gov't]. *Journal of Child Psychology & Psychiatry & Allied Disciplines, 60*(1), 91-99.

Martin, A., & Leslie, D. (2003). Psychiatric inpatient, outpatient, and medication utilization and costs among privately insured youths, 1997-2000. [Research Support, U.S. Gov't, P.H.S.]. *American Journal of Psychiatry, 160*(4), 757-764.

Mathew, A., Pettit, J., Lewinsohn, P., Seeley, J., & Roberts, R. (2011). Co-morbidity between major depressive disorder and anxiety disorders: shared etiology or direct causation? *Psychological medicine, 41*(10), 2023-2034.

Olino, T. M., Klein, D. N., Lewinsohn, P. M., Rohde, P., & Seeley, J. R. (2008). Longitudinal associations between depressive and anxiety disorders: A comparison of two trait models. *Psychological Medicine, 38*(3), 353-363.

Ormel, J., Raven, D., van Oort, F., Hartman, C., Reijneveld, S., Veenstra, R., et al. (2015). Mental health in Dutch adolescents: a TRAILS report on prevalence, severity, age of onset, continuity and co-morbidity of DSM disorders. *Psychological Medicine, 45*(2), 345-360.

Pella, J. E., Slade, E. P., Pikulski, P. J., & Ginsburg, G. S. (2020). Pediatric anxiety disorders: a cost of illness analysis. *Journal of Abnormal Child Psychology, 48*(4), 551-559.

Perez, R. G., Ezpeleta, L., & Domenech, J. M. (2007). Features associated with the non-participation and drop out by socially-at-risk children and adolescents in mental-health epidemiological studies. [Research Support, Non-U.S. Gov't]. *Social Psychiatry & Psychiatric Epidemiology, 42*(3), 251-258.

Ranoyen, I., Lydersen, S., Larose, T. L., Weidle, B., Skokauskas, N., Thomsen, P. H., et al. (2018). Developmental course of anxiety and depression from adolescence to young adulthood in a prospective Norwegian clinical cohort. *European Child & Adolescent Psychiatry, 27*(11), 1413-1423.

Ranta, K., La Greca, A. M., Kaltiala-Heino, R., & Marttunen, M. (2016). Social Phobia and Educational and Interpersonal Impairments in Adolescence: A Prospective Study. *Child Psychiatry & Human Development, 47*(4), 665-677.

Ranta, K., Vaananen, J., Frojd, S., Isomaa, R., Kaltiala-Heino, R., & Marttunen, M. (2017). Social phobia, depression and eating disorders during middle adolescence: longitudinal associations and treatment seeking. *Nordic Journal of Psychiatry, 71*(8), 605-613.

Reef, J., van Meurs, I., Verhulst, F. C., & van der Ende, J. (2010). Children's problems predict adults' DSM-IV disorders across 24 years. [Research Support, N.I.H., Extramural]. *Journal of the American Academy of Child & Adolescent Psychiatry, 49*(11), 1117-1124.

Rofey, D. L., Kolko, R. P., Iosif, A.-M., Silk, J. S., Bost, J. E., Feng, W., et al. (2009). A longitudinal study of childhood depression and anxiety in relation to weight gain. *Child psychiatry and human development, 40*(4), 517-526.

Shanahan, L., Copeland, W. E., Angold, A., Bondy, C. L., & Costello, E. J. (2014). Sleep problems predict and are predicted by generalized anxiety/depression and oppositional defiant disorder. [Research Support, N.I.H., Extramural Research Support, Non-U.S. Gov't]. *Journal of the American Academy of Child & Adolescent Psychiatry, 53*(5), 550-558.

Shevlin, M., McElroy, E., & Murphy, J. (2017). Homotypic and heterotypic psychopathological continuity: a child cohort study. *Social Psychiatry & Psychiatric Epidemiology, 52*(9), 1135-1145.

Sihvola, E., Keski-Rahkonen, A., Dick, D. M., Hoek, H. W., Raevuori, A., Rose, R. J., et al. (2009). Prospective associations of early-onset Axis I disorders with developing eating disorders. *Comprehensive psychiatry, 50*(1), 20-25.

Simon, E., Dirksen, C., Bogels, S., & Bodden, D. (2012). Cost-effectiveness of child-focused and parent-focused interventions in a child anxiety prevention program. *Journal of Anxiety Disorders\, 26*(2), 287-296\.

Sung, M., Erkanli, A., Angold, A., & Costello, E. J. (2004). Effects of age at first substance use and psychiatric comorbidity on the development of substance use disorders. [Comparative Study Research Support, Non-U.S. Gov't Research Support, U.S. Gov't, P.H.S.]. *Drug & Alcohol Dependence, 75*(3), 287-299.

Thirlwall, K., Cooper, P. J., Karalus, J., Voysey, M., Willetts, L., & Creswell, C. (2013). Treatment of child anxiety disorders via guided parent-delivered cognitive–behavioural therapy: Randomised controlled trial. *The British Journal of Psychiatry, 203*(6), 436-444.

Vaananen, J. M., Frojd, S., Ranta, K., Marttunen, M., Helminen, M., & Kaltiala-Heino, R. (2011). Relationship between social phobia and depression differs between boys and girls in mid-adolescence. [Research Support, Non-U.S. Gov't]. *Journal of Affective Disorders, 133*(1-2), 97-104.

Vaananen, J. M., Isomaa, R., Kaltiala-Heino, R., Frojd, S., Helminen, M., & Marttunen, M. (2014). Decrease in self-esteem mediates the association between symptoms of social phobia and depression in middle adolescence in a sex-specific manner: a 2-year follow-up of a prospective population cohort study. *BMC Psychiatry, 14*, 79.

van Steensel, F. J., Dirksen, C. D., & Bogels, S. M. (2013). A cost of illness study of children with high-functioning autism spectrum disorders and comorbid anxiety disorders as compared to clinically anxious and typically developing children. [Comparative Study]. *Journal of Autism & Developmental Disorders, 43*(12), 2878-2890.

Virtanen, S., Kuja-Halkola, R., Lundström, S., D’Onofrio, B. M., Larsson, H., Suvisaari, J., et al. (2021). Longitudinal associations of childhood internalizing psychopathology with substance misuse: A register-based twin and sibling study. *Journal of the American Academy of Child & Adolescent Psychiatry, 60*(5), 593-603.

Warner, L. A., Pottick, K. J., & Bilder, S. M. (2005). Clinical and Organizational Correlates of Medication for Youths in U.S. Mental Health Services. *Social Service Review, 79*(3), 454-481.

Wittchen, H. U., Frohlich, C., Behrendt, S., Gunther, A., Rehm, J., Zimmermann, P., et al. (2007). Cannabis use and cannabis use disorders and their relationship to mental disorders: a 10-year prospective-longitudinal community study in adolescents. [Research Support, N.I.H., Extramural Research Support, Non-U.S. Gov't]. *Drug & Alcohol Dependence, 88 Suppl 1*, S60-70.

Wolitzky-Taylor, K., Bobova, L., Zinbarg, R. E., Mineka, S., & Craske, M. G. (2012). Longitudinal investigation of the impact of anxiety and mood disorders in adolescence on subsequent substance use disorder onset and vice versa. [Multicenter Study Research Support, N.I.H., Extramural]. *Addictive Behaviors, 37*(8), 982-985.

Woodward, L. J., & Fergusson, D. M. (2001). Life course outcomes of young people with anxiety disorders in adolescence. [Research Support, Non-U.S. Gov't]. *Journal of the American Academy of Child & Adolescent Psychiatry, 40*(9), 1086-1093.

Wuthrich, V. M., Rapee, R. M., Cunningham, M. J., Lyneham, H. J., Hudson, J. L., & Schniering, C. A. (2012). A randomized controlled trial of the Cool Teens CD-ROM computerized program for adolescent anxiety. *Journal of the American Academy of Child & Adolescent Psychiatry, 51*(3), 261-270.

Yoshimasu, K., Barbaresi, W. J., Colligan, R. C., Voigt, R. G., Weaver, A. L., & Katusic, S. K. (2016). Mediating and Moderating Role of Depression, Conduct Disorder or Attention-Deficit/Hyperactivity Disorder in Developing Adolescent Substance Use Disorders: A Population-Based Study. *PLoS ONE [Electronic Resource], 11*(6), e0157488.

Zaider, T. I., Johnson, J. G., & Cockell, S. J. (2002). Psychiatric disorders associated with the onset and persistence of bulimia nervosa and binge eating disorder during adolescence. *Journal of Youth and Adolescence, 31*(5), 319-329.

**Appendix S6. Meta-analysis sensitivity analysis**

Anxiety outcomes

Figure S1: Association between childhood (≤18 years) anxiety disorder and subsequent childhood (≤18 years) anxiety disorder
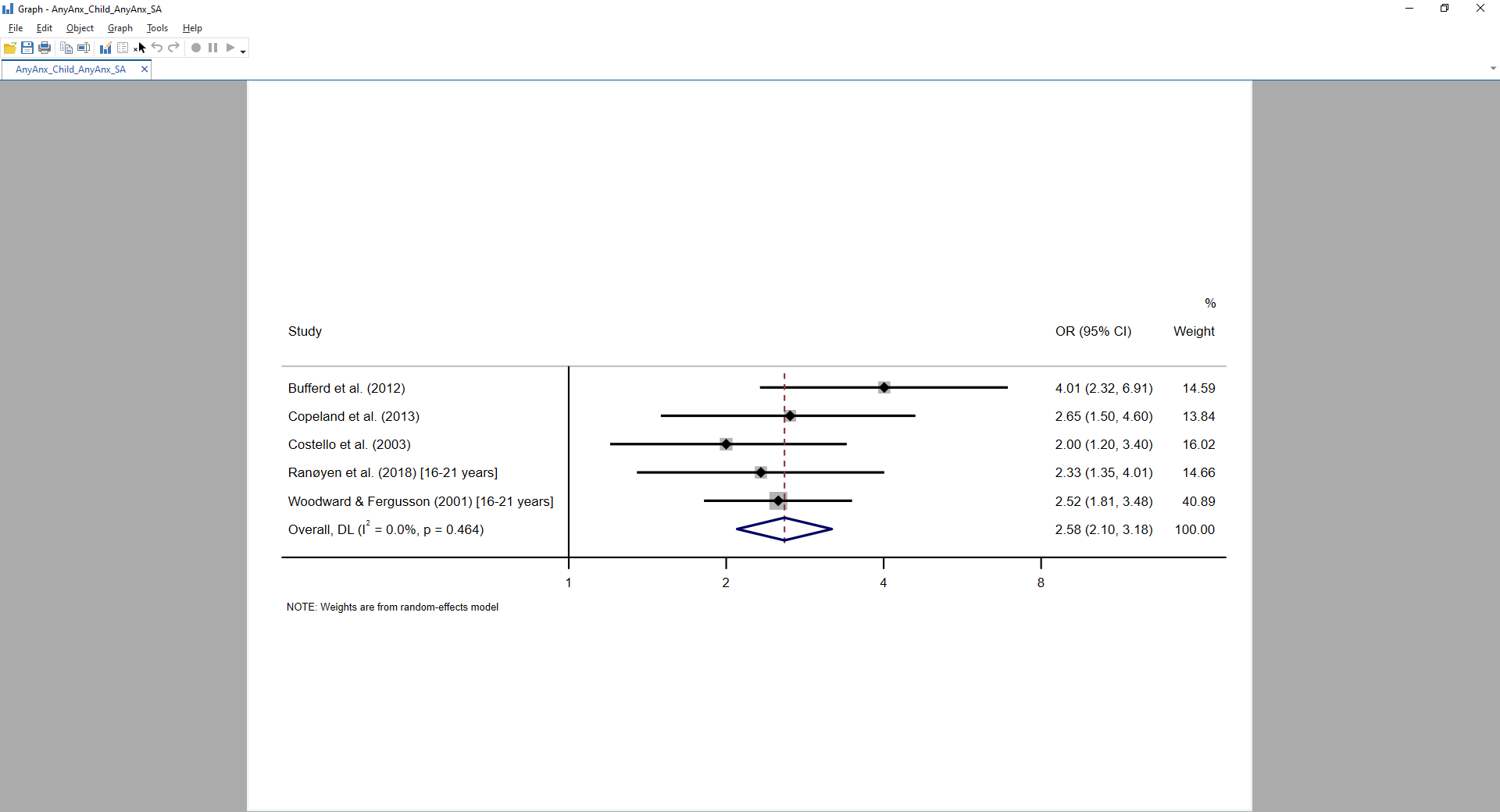


Figure S2: Association between childhood (≤18 years) separation anxiety disorder and subsequent childhood (≤18 years) agoraphobia


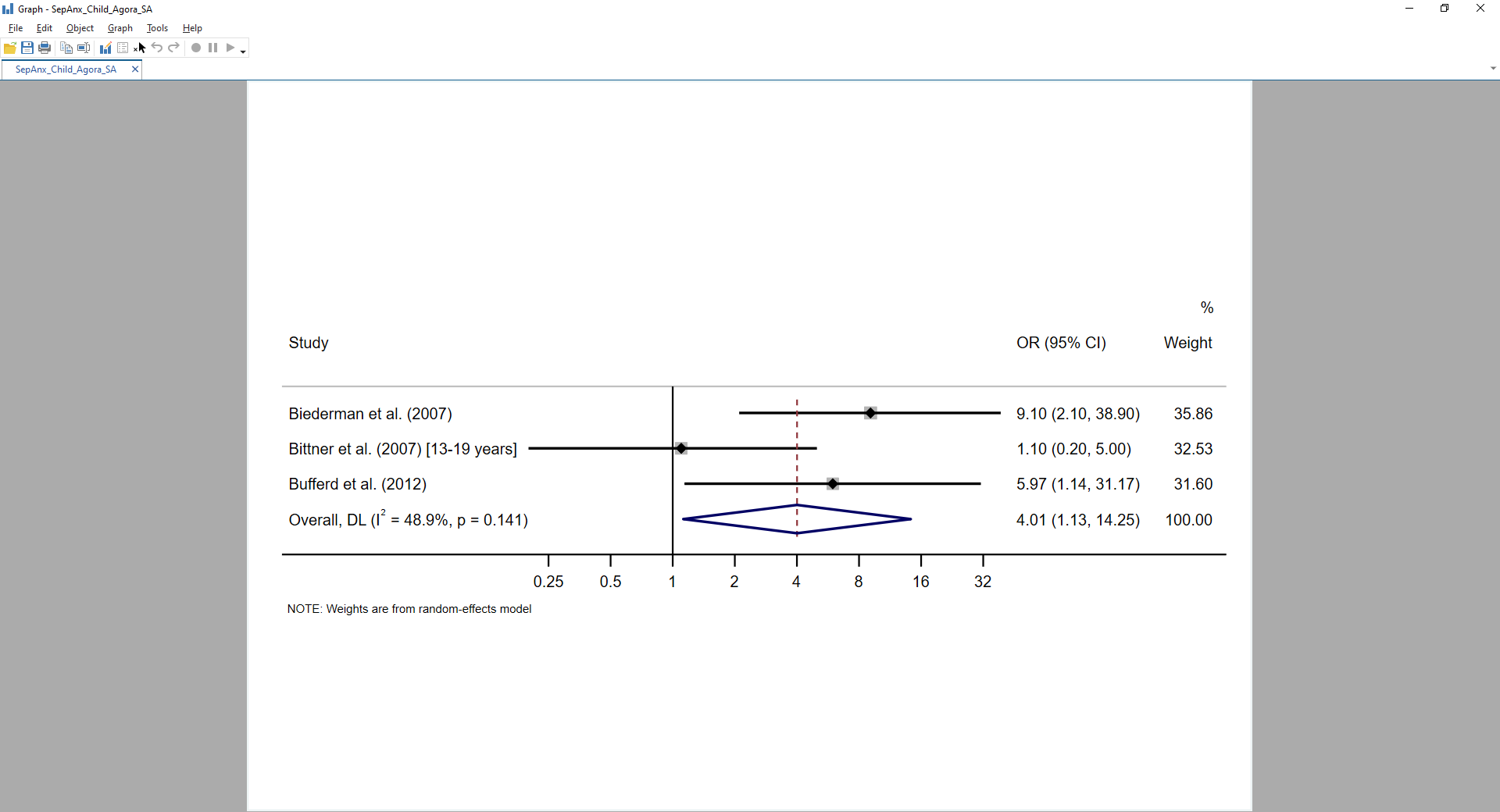


Figure S3: Association between childhood (≤18 years) social anxiety disorder and subsequent childhood (≤18 years) social anxiety disorder
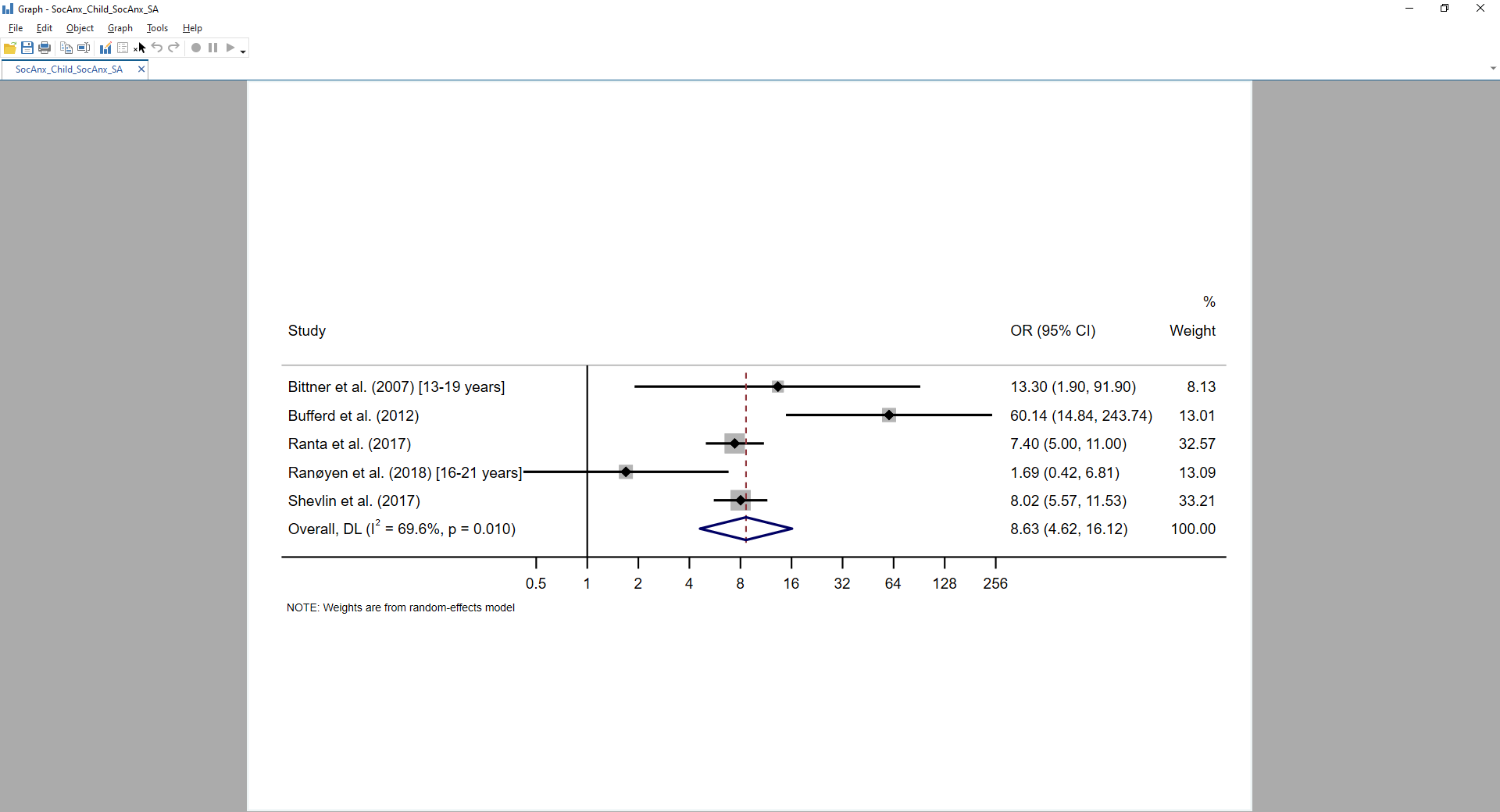


Mood outcomes

Figure S4: Association between childhood (≤18 years) anxiety disorder and subsequent childhood (≤18 years) depressive disorder


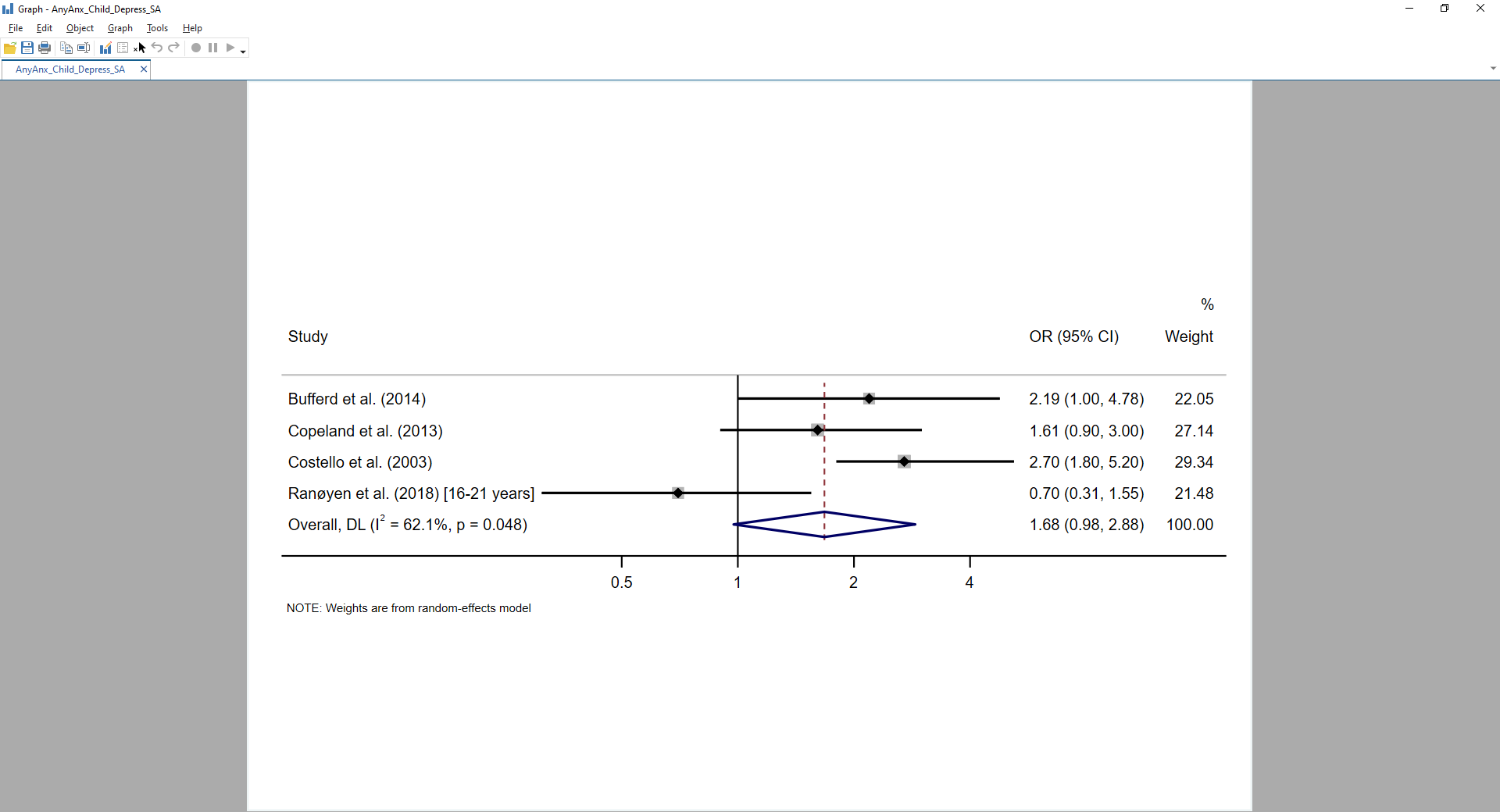


Substance outcomes

Figure S5: Association between childhood (≤18 years) anxiety disorder and subsequent childhood (≤18 years) substance use disorder
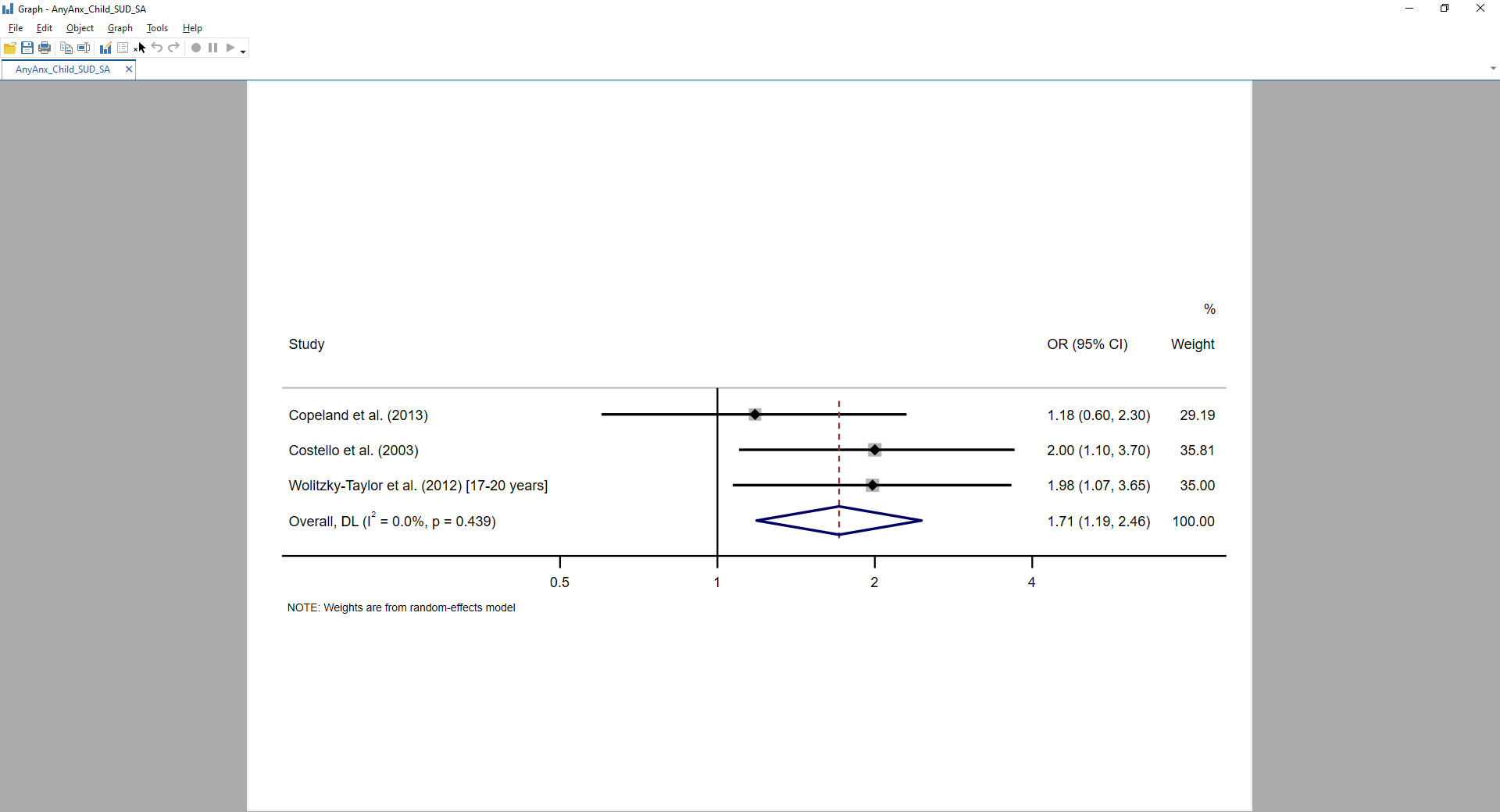

Supplement: Supplementary file 1 — Supplementary Material [file JCV2-3-e12149-s003.docx]
